# Potential Risk Factors for Aggression and Playfulness in Cats: Examination of a Pooling Fallacy Using Fe-BARQ as an Example

**DOI:** 10.3389/fvets.2020.545326

**Published:** 2021-01-05

**Authors:** Noema Gajdoš Kmecová, Barbara Pet'ková, Jana Kottferová, Rachel Sarah Wannell, Daniel Simon Mills

**Affiliations:** ^1^Department of Public Veterinary Medicine and Animal Welfare, Workplace of Applied Ethology and Professional Ethics, University of Veterinary Medicine and Pharmacy in Košice, Košice, Slovakia; ^2^Animal Behaviour, Cognition and Welfare Group, School of Life Sciences, University of Lincoln, Lincoln, United Kingdom

**Keywords:** behaviour, cat, C-BARQ, Fe-BARQ, pooling fallacy, hypothesis testing research, hypothesis generating research

## Abstract

Using a popular method of behaviour evaluation which rates the intensity of behaviour in different contexts, we demonstrate how pooling item scores relating to a given construct can reveal different potential risk factors for the dependent variable depending on how the total score is constructed. We highlight how similar simple total scores can be constructed through very different combinations of constituent items. We argue for the importance of examining individual item score distributions, and the results from different intensity thresholds before deciding on the preferred method for calculating a meaningful dependent variable. We consider simply pooling individual item scores which conflate context with intensity to calculate an average score and assuming this represents a biologically meaningful measure of trait intensity is a fallacy. Specifically using four items that describe intercat aggression and eleven that describe playfulness in cats in Fe-BARQ, we found sex and neuter status, social play and fearfulness were consistently significant predictors for intercat aggression scores; and age, age when obtained, social play and fearfulness were significant predictors of playfulness scores. However, the significance of other factors such as scratching varied with the threshold used to calculate to the total score. We argue that some of these inconsistent variables may be biologically and clinically important and should not be considered random error. Instead they need to be evaluated in the context of other available evidence.

## Introduction

Surveys of animal carers allow us to potentially gather large datasets on the individuals with which they are familiar. These reports can be both reliable and valid, with the latent structure showing convergence with other more objective measures of the same construct e.g., Wright et al. (1). The structure of such instruments is often revealed by Principal Components Analysis (PCA), which tells us that the items are related. However, when pooling scores of individual items to create total or average scores of the components, we need to consider carefully what this value represents. For example, if the items relate to the occurrence of the behaviour in a given context and are scored in a binary way (present-absent), then the sum score of items indicates the number of contexts in which the behaviour occurs; but if individual items relate to the frequency or intensity of the behaviour in a given context (as often occurs with Likert scales) then the interpretation of the sum/average of all items is more complicated. The Feline Behavioural Assessment and Research Questionnaire (Fe-BARQ) (2, 3) is a potentially useful behavioural instrument that was developed for the purposes of quantitative behavioural evaluation of pet cats in their home environment. It comprises of 85 items which group into 23 factors measuring different aspects of cat behaviour (e.g., playfulness, sociability with people, purring and prey interest); this includes some potentially problematic issues such as aggressive behaviour (towards people, other cats and dogs) and repetitive behaviours. It also includes 21 other “miscellaneous” items which are not part of any of the preceding factors but which might be important to consider from the cat-owner relationship perspective (e.g., excessive vocalisation, spraying, playing “fetch,” fearfulness or inappropriate scratching). Each item relates to a behaviour, often in some specific context, and can be scored using a Likert scale referring to the frequency of behaviour occurrence, where 0 = never, 1 = seldom, 2 = sometimes, 3 = usually and 4 = always (3). Four items make up the score for the factor “aggression towards other cats,” with each item relating to the frequency of the behaviour in four different contexts. Aggression in cats can be context specific (4) whether directed towards other cat e.g., Lindell et al. (5) and Levine et al. (6) or human e.g., Chapman and Voith (7) and Ramos and Mills (8). Accordingly, cats with a total factor score of 4 for aggression towards other cats could be scoring low (1) in each of the four contexts, or they could be scoring high (4) on one item and zero on the other three i.e., always showing an aggressive response in one specific context but never in the other three. These are very different behaviour profiles and clinical behavioural implications; they may also have serious implications when trying to investigate relationships between survey scores and potential risk factors. Factor scores may be strongly influenced by extreme values and while it may be argued that this reflects the intensity of a trait in some sense, it may not be very accurate. This is of particular concern when we are interested in risk factors for a given factor especially when it relates to a potentially complex problem such as aggressive behaviour (9, 10). Unfortunately, without access to the raw data of studies, we have no way of knowing the extent to which pooled results (and the conclusions which are drawn as a result) are affected by this potential problem.

One way to potentially address this is to convert the individual constituent Likert scales making up a factor into binary outcomes so that the total reflects the extent of the behaviour occurring at a certain level across the contexts described. This intensity could be simply presence/absence of the behaviour in a traditional 1-0 behaviour sampling sense [as done by (11)]. However, in some contexts it might be useful to consider a higher severity threshold, for example the threshold might be raised based on what are considered normal levels of expression or some other threshold of interest. This could be determined *post-hoc* from the distribution of the raw data if it is recognised that the research is fundamentally at an exploratory stage, without strong evidence to support the testing of specific hypotheses (12). Alternatively, a threshold could be set based on other factors, such as the point at which the behaviour has been shown to become a concern. The question then arises: What is the impact of cutting the data to create this new binary outcome at different levels? In this paper we illustrate and explore the implications of this potentially significant, but seemingly largely overlooked, phenomenon (10, 13, 14) by examining the potential risk factors revealed for various aspects of feline social behaviour using the recently developed Fe-BARQ scale which, like its canine equivalent (Canine Behaviour and Research Questionnaire, C-BARQ) is potentially subject to such misuse.

So far, Fe-BARQ has only been used to study associations between breed, coat type, eye colour and behaviour of cats (2). By contrast, C-BARQ, which uses a similar scoring system, has been utilised in a much wider range of studies and appears to be becoming a popular instrument for investigating increasingly complex relationships beyond the sort of behaviour profiling for which it was originally developed (10, 15). For example scores have been used to make inferences about welfare (14) and to inform working dog selection (16) but also to make heritability estimates for a range of behaviours (17) and to investigate more closely the potential risk factors of specific issues such as aggressive behaviour (9, 18, 19). It is in these latter contexts that the issues relating to the pooling of scores of similar value are of potential concern and we suggest a fallacy. The diversification in the use of this scale make our current work very timely.

In this paper, our primary aim is to illustrate the effect of this “pooling fallacy” in relation to our own dataset for Fe-BARQ, although the issue applies to any similarly scored instrument, such as C-BARQ. We illustrate this by reference to two behavioural elements within the Fe-BARQ made up of quite different numbers of items (aggressiveness towards other cats and non-social playfulness). Since there are more ways to potentially calculate non-extreme values when there are more items, we hypothesise that there will be greater variability in the risk factors revealed using different forms of average score calculation when there are more items making up the factor. Specifically, if we convert Likert scale data into binary outcomes with different thresholds, we may not only reveal different significant associations with demographic variables but also that these relationships may be different from those identified using the average score of all items making up that factor (*Averaged total score*).

Within Fe-BARQ, playfulness has the highest number of constituent items (14), and so we used a subset of 11 items within this factor clearly related to object/self play in cats as our primary focus. By contrast, aggressive behaviour towards familiar cats is calculated on the basis of only 4 items. The choice of these two features not only allows us to illustrate the breadth of the issue, but also the potential impact on two features of cat behaviour used to draw opposing conclusions about their welfare: i.e., positive in the case of play (20, 21) and negative in the case of signs of social stress leading to intercat aggressive behaviour (22–24).

As a secondary aim we examine the results generated by the different methods with regards to their interpretation as potential risk factors. We recognise that no single method is without its limitations, and so emphasise the importance of careful consideration of the nature of the research being undertaken. A key feature in this regard (and in the choice of statistical threshold conventions) is a clear distinction between research that seeks to primarily generate hypotheses for later investigation (such as the analysis of potential risk factors) and research which is focused on testing and differentiating between specific hypotheses (12). It is worth pointing out that both forms of research can involve statistical testing of hypotheses, but the results reduce scientific uncertainty to different degrees because of their methodological rigour. Within hypothesis generating research we primarily reveal what might be important, while within hypothesis testing research we focus on specifically excluding competing hypotheses. Within hypothesis generating research, since we are often exploring what the data reveals, it can be useful to consider and contrast the outcome from different methodologies to see what is found consistently to be significant (25). However, researchers will often only report the outcome of a single test. Therefore, as part of our second aim, we consider first how examination of consistency of results across methods of calculation can be useful in prioritising factors for subsequent consideration. We then consider how examination of the correlation between the average score of all items compared to the average score of the data converted into binary outcomes at different levels can provide useful insight into the nature of the factor being explored.

## Materials and Methods

### Survey Structure and Distribution

An online English survey using Fe-BARQ was developed using Google forms. It consisted of 27 demographic questions about the cat and cat ownership. The second part of the survey consisted of 100 items concerning cat behaviour including 85 from Fe-BARQ, which were subdivided into 23 sections [as per (3)] with an additional 15 items included in a “miscellaneous” section (as per the recommendation of instrument's author—personal communication, 2017).

The survey was launched in December 2017, publicised *via* social media (e.g., Facebook) and closed in February 2018 (For the online survey as presented *via* Google forms see Supplementary Material—Questionnaire).

### Creation of Dataset for Analysis

For the purposes of this study, we focus only on the results relating to aggressive behaviour towards familiar cats (hereon referred to simply as “aggression”) and object/self play (hereon referred to simply as “playfulness” — social play was excluded at this point due to its confusion with aggressive behaviour—this point is discussed further below) as dependent variables. Eleven of the 27 demographic questions about the cat and cat ownership were relevant potential predictors of these responses (Table 1). In addition, and based on the clinical behaviour literature (8, 26–29), social play (playing with other household cats), inappropriate scratching [scratches claws on inappropriate objects or surfaces indoors (furniture, rugs, curtains, wallpaper, etc.)], spraying [sprays (standing position with tail raised vertically) outside of the litter box or on other surfaces and objects (e.g., furniture, walls, people's legs, etc.) indoors] and fear of noises [runs and/or hides in response to sudden or loud noise (e.g., vacuum cleaner, car backfire, road drills, dropped object, sounds of musical instruments, doorbells or someone knocking on the door)] were also considered as potential predictors for the analysis of the aggression and playfulness scores.

**Table 1:** Demographic data on selected questions (*N* = 1,805).

**Age (years)**	***N***	**%**	**Age when obtained**	***N***	**%**
6 months−1 year	113	6.26	Unweaned kitten (0–2 months)	332	18.39
1	179	9.92	Kitten (2–6 months)	1,042	57.73
2	228	12.63	Junior (6 months−2 years)	282	15.62
3	239	13.24	Mature (3–10years)	137	7.59
4	173	9.58	Senior (11+ years)	12	0.66
5	158	8.75	**Lifestyle**		
6	135	7.48	Indoors only	821	45.48
7	100	5.54	Indoors with controlled or limited access to space outdoors (e.g., fenced garden or pen)	494	27.37
8	82	4.54	Indoors with free access outdoors	481	26.65
9	70	3.88	Lives in pen/stall/cage and has controlled access outdoors	2	0.11
10	75	4.16	Outside only (no access indoors)	7	0.39
Over 11	253	14.02	**Left alone per week**		
**Sex and neutered status**			0–6 h	720	39.89
Neutered female	854	47.31	7–15 h	190	10.53
Non-neutered female	59	3.27	16–29 h	450	24.93
Neutered male	844	46.76	30–42 h	360	19.94
Non-neutered male	48	2.66	43 and more hours	85	4.71
**Breed type**			**Is this cat currently suffering from any health problems?**		
Domestic long hair/mixed breed	275	15.24	No	1,459	80.83
Domestic short hair/mixed breed	1,046	57.95	Yes	346	19.17
Mix of pure breeds (or if only one parent of your cat is pedigree)	105	5.82	**Does a dog live in your household?**		
Pure breed (Pedigree)	379	21.00	No	1,180	65.37
**Obtained from**			Yes	625	34.63
Breeding cattery	268	14.85			
Cattery (boarding/foster)	29	1.61			
Friend/relative/neighbour	425	23.55			
Home (born at)	98	5.43			
Pet store (purchase)	42	2.33			
Pet store (rescue)	73	4.04			
Selling website	100	5.54			
Shelter	427	23.66			
Street (as a stray)	303	16.79			
Veterinary hospital	40	2.22			

Data were initially cleaned, by checking for and removing duplicates and then for the purposes of this study only datasets relating to multicat households were selected for analysis (since we wanted to include intercat play as a potential independent variable for both dependent variables). Finally, those with any incomplete data representing unknown or absent responses were removed from our sample population.

Two demographic items [In a typical week, how many days is this cat left alone (no people around) at home for more than 1 h; On a typical day, how many hours is this cat left alone (no people around) at home?] were subsequently combined into a single composite variable by multiplying days left alone per week by hours left alone per day (using the midpoint value of the range within each category of answer option for both items, e.g., for answer option: 1–3 days left alone per week, midpoint value used to calculate the composite variable was “2”). The resulting value then represented time left alone per week and these results were then ordered into five different categories (up to 42 h [based on (3)] creating a new variable: “time left alone per week” (henceforth described as “left alone”). In addition, the two items on sex and neuter status were combined into one new variable “sex and neuter status” with four categories (female non-neutered, female neutered, male non-neutered, male neutered), before analysis.

Initially we calculated descriptive summary statistics, and examined the frequency distribution for both the *Averaged total scores* [the sum of scores for the factor divided by the number of items making up that factor as per the recommendation of (3)] and specific item scores. Aggression scores were made up of four items [as per (3)], whereas for the playfulness score we based our scores on only the 11 items relating to object, predatory or self play. We excluded three of the items included in the original Fe-BARQ [Chases and ambushes other household members (including pets) playfully; Initiates mutual chasing by running from room to room in the house; and Plays with other household cat(s)] as these items might relate to rough and tumble social play, which it has been argued has a different neuro-affective basis [PLAY *sensu* (30)] and might also be confused with forms of agonistic behaviour resulting in related outcomes and thus confounded correlates. By doing this, it can also be argued that the remaining 11 items are related to a single affective system [SEEKING *sensu* (30)].

We then converted individual item response scores into binary data based on different thresholds (1, 2, 3 or 4, i.e., a cut off threshold of 2 means scores of “0 = never” or “1 = seldom” were recoded as 0, while scores of “2 = sometimes”, “3 = usually” or “4 = always” were recoded as 1). These were then summed and divided by the number of items to create a standardised score between 0 and 1. These new variables are henceforth referred to as *Averaged total binary scores 1, 2, 3* or *4* respectively.

### Data Analysis

In order to examine the impact of different ways of calculating the total score on the significance of a range of potential risk factors, we initially used non-parametric tests (Mann-Whitney test, Kruskal-Wallis test, Kendall's Tau correlation coefficient). These examined the relationship between the *Averaged total scores* and *Averaged total binary scores* for both aggression and playfulness and the following demographic factors: age (by year up to age 11+ years of age), sex including neuter status, breed type [domestic shorthair/mixed breed, domestic longhair/mixed breed, purebreed (pedigree), mix of pure breeds or if only one parent of your cat is pedigree], source of animal [Home (born at), Friend/relative/neighbour, Veterinary hospital, Street (as a stray), Shelter, Breeding cattery, Cattery (boarding/foster), Pet store (purchase), Pet store (rescue), Selling website], age when obtained [unweaned kitten (0–2 months), kitten (2–6 months), junior (6 months−2 years), mature (3–10 years), senior (11+ years)], lifestyle [Indoors only, Indoors with controlled or limited access to space outdoors (e.g., fenced garden or pen), Indoors with free access outdoors, Outside only (no access indoors), Lives in pen/stall/cage and has controlled access outdoor], time left alone per week (0–6 h, 7–15 h, 16–29 h, 30–42 h, 43 h and more), health status (healthy or not) and presence of a dog in the home (present or not). For the overview of demographic factors see Table 1.We also examined the relationship between scores and items relating to the single Fe-BARQ item most clearly related to social play [Plays with other household cat(s)], inappropriate scratching, spraying and fearfulness.

A selection of the demographic items from the univariate analysis (see sections **Results**, **Aggression** and **Playfulness**) was then used in a backward stepwise procedure to generate minimal adequate models of the relationship between the independent variables and the two behaviour factor scores (various forms of score for playfulness and aggression). Only items found to be significant at *p* < 0.05 in the univariate analysis were initially included in the model and non-significant factors (*p* ≥ 0.05) in the multivariate analysis were serially removed until the final model contained only significant factors (interactions were not included). Assumptions concerning homogeneity of variance and heteroscedasticity for these models were evaluated from visual inspection of the standardised residual plots and deemed to be acceptable.

In order to consider the implications of cutting the data relating to aggression and playfulness at different levels of score, we not only examined the data distribution, but also calculated Kendall's Tau for the association between the scores of items making up that component and total scores. Kendall's tau was used as it is more mathematically tractable to the large number of ties present (31).

All statistical analyses were conducted using IBM SPSS ver. 25.0.0.1.

### Ethics Statement

All experiments (the questionnaire survey and its analysis) were performed in accordance with guidelines and regulations of University of Veterinary Medicine and Pharmacy in Košice. The experimental protocol used in this study (internet questionnaire survey) did not require approval by the local Ethics committee of the University of Veterinary Medicine and Pharmacy in Košice for handling of animals. As the online completed survey used in this study was directed at owners, it was assumed that all participants were over 18 and participants' consent was inferred from the completion of the survey and submission of the data. No sensitive information was stored from any of the participants and any incomplete surveys, which might indicate withdrawal of consent, were not used.

## Results

### Sample Characteristics

5,631 responses were received for the online survey, this dropped to 5,353 after removing duplicates, of which 3,681 (68.7%) related to multicat households. After removal of those with incomplete/unknown item responses, data relating to 1,805 cats were included in the analysis.

Mean age of the sample population was 5.1 years (±3.5) with 47.3% neutered females, 3.3% non-neutered females, 46.8% neutered males and 2.7% of intact males. The majority (73.2%) was non-pedigree (mix breed) domestic shorthair and longhair cats, with most (78.9%) obtained from a breeding cattery, friend/relative/neighbour, shelter or street as a stray cats and more than half (57.8%) between the ages of 2–6 months. Almost half of the cats (45.5%) were kept strictly indoors. Almost 40% of cats (39.9%) were left alone up to 6 h per week and <5% of cats (4.7%) were left alone more than 43 h per week; most of the cats were reported to be currently healthy (80.8%). About two thirds of cats (65.37%) lived without a dog in the home. Further details on the demographics are provided in Table 1.

Descriptive summary scores for aggression and playfulness are shown in Tables 2, 3, below, respectively. Item scores were not normally distributed (frequency distribution histograms per each item and score calculation method for aggression and playfulness can be found as Supplementary Graphs 1–25).

**Table 2:** Descriptive summary data for the aggression factor: individual items, averaged total score and averaged total binary scores 1,2,3,4 of the four individual items making up this factor, Kendall's Tau describes correlation between averaged total score and each item and binary score in the table, all correlations are significant at 0.01 level (2-tailed).

**Aggression**	**Median**	**Mean**	**Min**.	**Max**.	**25% percentile**	**75% percentile**	**Kendall's tau**
44. Growls/hisses when approached by a familiar (household) cat while eating.	0	0.64	0	4.00	0	1.00	0.673
45. Growls/hisses when approached by a familiar (household) cat at a favourite resting place.	0	0.68	0	4.00	0	1.00	0.742
46. Growls/hisses when stared at, growled or hissed at by a familiar (household) cat.	0	0.82	0	4.00	0	1.00	0.790
47. Attacks (scratches/bites/attempts to bite) when stared at, growled or hissed at by a familiar (household) cat.	0	0.73	0	4.00	0	1.00	0.722
Averaged total score	0.25	0.72	0	4.00	0	1.00	-
Averaged total binary score 1	0.25	0.36	0	1.00	0	0.75	0.916
Averaged total binary score 2	0	0.21	0	1.00	0	0.25	0.807
Averaged total binary score 3	0	0.10	0	1.00	0	0	0.616
Averaged total binary score 4	0	0.04	0	1.00	0	0	0.429

*Min., miminum value, Max., maximum value, Fe-BARQ scores for answer options: 0 = never, 1 = seldom, 2 = sometimes, 3 = usually, 4 = always, Averaged total binary score 1: 0 = 0 and 1,2,3,4 = 1; Averaged total binary score 2: 0,1 = 0 and 2,3,4 = 1; Averaged total binary score 3: 0,1,2 = 0 and 3,4 = 1; Averaged total binary score 4: 0, 1,2,3 = 0 and 4 = 4*.

**Table 3:** Descriptive summary data for playfulness items: individual items, averaged total score and averaged total binary scores 1,2,3,4 of 11 individual items above, and correlation between averaged total score and each item and binary score in the table (Kendall's Tau coefficient), all correlations are significant at 0.01 level (2-tailed).

**Playfulness**	**Median**	**Mean**	**Min**.	**Max**.	**25% percentile**	**75% percentile**	**Kendall's tau**
1. Quickly learns how to play with new introduced toys.	3.00	3.09	0	4.00	3.00	4.00	0.538
2. Curious: actively investigates/explores new objects, sights, or changes in its environment.	4.00	3.34	0	4.00	3.00	4.00	0.466
3. Carries small objects/toys in the mouth to interact with.	2.00	2.07	0	4.00	1.00	3.00	0.574
4. Runs and jumps in the air.	3.00	2.48	0	4.00	2.00	3.50	0.668
5. Engages in active jumping and climbing on high surfaces, furniture or curtains.	3.00	2.89	0	4.00	2.00	4.00	0.535
6. Exhibits sudden bursts of running or climbing in certain periods of the day.	3.00	2.80	0	4.00	2.00	4.00	0.593
7. Exhibits sudden jumping and running during playful activity.	3.00	2.76	0	4.00	2.00	4.00	0.654
8. Stalks, chases or pounces on moving objects (string, balls, soft toys, etc.) during playful activity.	3.00	3.07	0	4.00	2.00	4.00	0.619
9. Displays running/chasing and hunting/pouncing on unseen/imaginary prey/objects.	2.00	2.24	0	4.00	1.00	3.00	0.597
11. Chases or follows shadows or light spots.	3.00	2.41	0	4.00	1.00	4.00	0.528
13. Initiates interactive play with people in the home (i.e., brings toys, strings, or small objects to play with).	1.00	1.65	0	4.00	0	3.00	0.578
Averaged total score	2.72	2.62	0	4.00	2.09	3.27	-
Averaged total binary score 1	1.00	0.90	0	1.00	0.91	1.00	0.596
Averaged total binary score 2	0.81	0.77	0	1.00	0.64	1.00	0.779
Averaged total binary score 3	0.63	0.60	0	1.00	0.36	0.82	0.857
Averaged total binary score 4	0.27	0.34	0	1.00	0.09	0.55	0.764

*Min., minimum value, Max, maximum value, Fe-BARQ scores for answer options: 0 = never, 1 = seldom, 2 = sometimes, 3 = usually, 4 = always, Averaged total binary score 1: 0 = 0 and 1,2,3,4 = 1; Averaged total binary score 2: 0,1 = 0 and 2,3,4 = 1; Averaged total binary score 3: 0,1,2 = 0 and 3,4 = 1; Averaged total binary score 4: 0,1,2,3 = 0 and 4 = 1*.

For aggression (Table 2), the median value of the constituent items was 0 in all cases; Kendall's tau revealed reasonably similar correlations between these individual items and average factor scores (*Averaged total score)*. Much greater variation was observed between *Averaged total score*, and *Averaged total binary scores*, with correlation reducing the higher the threshold.

The median for the 11 items making up the factor playfulness (Table 3) ranged from 1 to 4 with 3 the most frequent value (7/11 questions). Correlation coefficients between each item and average factor score was more variable than with aggression; the trend for weaker correlation between *Averaged total binary scores* and *Averaged total score* with higher binary cut off points seen with aggression was not apparent (Table 3).

### Potential Risk Factors for Behavioural Outcomes

The significant predictors for each factor differed according to the method used for calculating total score.

#### Aggression

For *Averaged total score*, univariate analysis identified 11 significant associations (*p* ≤ 0.05): age, breed type, sex and neuter status, source of animal, age when obtained, lifestyle, health, social play, fearfulness, scratching, and spraying (details of univariate analysis results for aggression are available in Supplementary Tables 1–3). Seven of these remained in the minimal adequate model: age, breed type, sex and neuter status, social play, fearfulness, scratching and spraying.

For *Averaged total binary score 1*, univariate level of analysis revealed 10 of the same items as found for the *Averaged total score* to be significant, but scratching was excluded. Likewise, the minimum adequate model for this variable included only six items (Table 4).

**Table 4:** F and partial eta squared value for minimal adequate models for different ways of aggression score calculation methods, ^*^p ≤ 0.05, ^**^p ≤ 0.01, ^***^p ≤ 0.001.

**Aggression- minimal adequate model**	**Averaged total score**		**Averaged total binary score 1**		**Averaged total binary score 2**		**Averaged total binary score 3**		**Averaged total binary score 4**	
	**F**	**Partial eta squared**	**F**	**Partial eta squared**	**F**	**Partial eta squared**	**F**	**Partial eta squared**	**F**	**Partial eta squared**
Age	4.381*	0.002	26.629***	0.015	x	x	x	x	x	x
Breed type	2.963*	0.005	3.960**	0.007	x	x	x	x	x	x
**Sex and neuter status**	17.575***	0.029	16.141***	0.026	13.935***	0.023	10.574***	0.017	6.107***	0.010
**Q Social play**	239.162***	0.118	134.753***	0.070	280.232***	0.135	240.549***	0.118	141.570***	0.073
**Q Fearfulness**	7.061**	0.004	7.267**	0.004	7.798**	0.004	4.650*	0.003	4.041*	0.002
Q Scratching	15.257***	0.008	x	x	17.022***	0.009	14.101***	0.008	7.746**	0.004
Q Spraying	14.251***	0.008	17.736***	0.010	11.426***	0.006	7.280**	0.004	x	x
*R^2^*	0.226		0.192		0.189		0.161		0.097	
*Adj. R^2^*	0.221		0.188		0.186		0.157		0.094	

*Bold items are significant across all models*.

For *Averaged total binary score 2*, univariate analysis 10 same items as found for *Averaged total score* were significant with health excluded: age, breed type, sex and neuter status, source of animal, age when obtained, lifestyle, social play, fearfulness, scratching and spraying. Five of the items identified when trying to predict the *Averaged total score* (including scratching) were retained in the minimal adequate model (Table 4).

For *Averaged total binary score 3*, univariate analysis revealed 10 significant items, same as for *Averaged total score* but with age when obtained excluded, namely age, breed type, sex and neuter status, source of animal, lifestyle, health, social play, fearfulness, scratching and spraying. The same five items identified in the previous model when trying to predict the *Averaged total binary score 2* were retained in the minimal adequate model (Table 4).

For *Averaged total binary score 4*, (i.e., when only aggression that was rated as always occurring in the given context was used to calculate the score), univariate analysis revealed five significant items (with age, source of animal, age when obtained, lifestyle, health and spraying excluded when compared with *Averaged total score*), namely: breed type, sex and neuter status, social play, fearfulness and scratching; four of which were retained in the minimal adequate model (Table 4).

Across these analyses, only three items: sex including neuter status, social play and fearfulness were consistent predictors of the dependent variable, which was always some form of severity of aggression. Neutered females consistently had the highest score for aggression, followed by entire females and neutered males, (Supplementary Tables 1–3 for results of univariate analysis) with entire males least aggressive except when using the *Averaged total binary score 4* method, where they were replaced by neutered males (Supplementary Table 2). The item on social play correlated negatively but weakly (using conventional thresholds for interpretation) with aggression using each calculation method (Kendall's tau *r* = −0.306 to −0.208, *p* ≤ 0.001) (see Table 5), while fearfulness correlated significantly but very weakly (Kendall's tau *r* = 0.079–0.066, *p* ≤ 0.001) (Supplementary Table 3).

**Table 5:** F and partial eta squared values for minimal adequate models for different ways of playfulness score calculation methods,^*^p ≤ 0.05, ^**^p ≤ 0.01, ^***^p ≤ 0.001.

**Playfulness- minimal adequate model**	**Averaged total score**		**Averaged total binary score 1**		**Averaged total binary score 2**		**Averaged total binary score 3**		**Averaged total binary score 4**	
	**F**	**Partial eta squared**	**F**	**Partial eta squared**	**F**	**Partial eta squared**	**F**	**Partial eta squared**	**F**	**Partial eta squared**
**Age**	111.377***	0.059	28.401***	0.016	97.984***	0.052	114.713***	0.060	66.932***	0.036
Breed type	10.350***	0.017	x	x	6.249***	0.010	8.354***	0.014	11.643***	0.019
Sex and neuter status	2.736*	0.005	x	x	2.954*	0.005	x	x	4.062**	0.007
Obtained from	x	x	2.132*	0.011	x	x	x	x	x	x
**Age when obtained**	8.642**	0.005	7.779**	0.004	4.469*	0.002	6.416*	0.004	5.151*	0.003
Lifestyle	2.813*	0.006	2.714*	0.006	x	x	x	x	x	x
Left alone	7.474**	0.004	x	x	x	x	5.970*	0.003	8.436**	0.005
Dog	9.448**	0.005	8.607**	0.005	9.407**	0.005	8.811**	0.005	x	x
Health	9.880**	0.006	13.263***	0.007	9.723**	0.005	11.245***	0.006	x	x
**Q Social play**	462.735***	0.206	250.154***	0.123	380.807***	0.175	383.847***	0.176	312.209***	0.148
**Q Fearfulness**	11.045***	0.006	4.235*	0.002	4.900*	0.003	6.882**	0.004	8.428**	0.005
Q Scratching	5.898*	0.003	x	x	x	x	3.914*	0.002	4.231*	0.002
*R^2^*	0.435		0.272		0.363		0.382		0.315	
*Adj. R^2^*	0.430		0.264		0.358		0.378		0.310	

*Bold items are common to all models*.

#### Playfulness

The type of effects (different predictors significant depending on total score calculation method used) observed in relation to the aggression factor were even more extensive with the playfulness factor which was comprised of 11 items (Table 5).

For *Averaged total score*, univariate analysis identified 12 significant predictors (*p* ≤ 0.05): age, breed type, sex and neuter status, source of animal, age when obtained, lifestyle, left alone, dog, health, social play, fearfulness and scratching (details of univariate analysis results are available in Supplementary Tables 4–6). Eleven of these remained in the minimal adequate model, with source of animal (where it was obtained from) being excluded (Table 5).

For *Averaged total binary score 1*, univariate analysis revealed 11 of the same items as found for the *Averaged total score* to be significant: age, breed type, sex and neuter status, source of animal, age when obtained, lifestyle, left alone, dog, health, social play and fearfulness with scratching excluded. The minimal adequate model for this variable included 8 items: age, source of animal, age when obtained, lifestyle, dog, health, social play and fearfulness (Table 5); the source of the animal was retained in this case unlike in the multivariate model for predicting *Averaged total score*.

For *Averaged total binary score 2*, univariate analysis revealed the same 11 items as for *Averaged total binary score 1* were significant. Eight items were retained in the minimal adequate model, 6 of which were common with the model generated for predicting *Averaged total binary score 1*, and all were common with 8 of the 11 retained to predict *Averaged total score* (Table 5).

For *Averaged total binary score 3*, univariate analysis revealed the same 12 significant items as for *Averaged total score*. Nine items were retained in the minimal adequate model, of which 7 were common with the model generated for predicting *Averaged total binary score 2;* 5 with the model generated for predicting *Averaged total binary score 1* and all were common with 9 of the 11 retained to predict *Averaged total score* (Table 5).

For *Averaged total binary score 4*, (when only playfulness that was rated as always occurring in the given context was used to calculate the score, univariate analysis revealed the same 12 significant items as found to be significant for the *Averaged total score*. Eight items were retained in the minimal adequate model. Seven were common to the predictors retained to calculate *Averaged total binary score 3*, 6 with the model generated for predicting *Averaged total binary score 2*; 4 with the model generated for predicting *Averaged total binary score 1* and all were common with 8 of the 11 retained to predict *Averaged total score* (Table 5).

Thus, between 8 and 11 potentially significant predictors for playfulness were identified using simple non-parametric statistics, according to the factor score calculation method, but only age, age when obtained, social play and fearfulness remained significant predictors across all methods of calculating the dependent variable score for all minimal adequate models.

There was a consistent significant effect of age on playfulness scores (*H* = 195.676–399.761, *p* ≤ 0.001, Supplementary Tables 4, 5) with playfulness scores reducing with age regardless of the calculation method. There were also consistent effects of age when obtained on playfulness scores (*H* = 48.036–84.260, p ≤ 0.001, Supplementary Tables 4, 5). Cats obtained as kittens (between 2 and 6 months of age) consistently had the highest scores for playfulness, and those obtained as seniors (11 years and older) were least playful (Supplementary Table 5). The correlation between playfulness and social play scores were consistent but moderate-weak (Kendall's tau *r* = 0.459–0.377, *p* ≤ 0.001, Supplementary Table 6). Fearfulness scores correlated consistently but very weakly negatively with playfulness scores (Kendall's tau *r* = −0.073 to −0.063, *p* ≤ 0.001, Supplementary Table 6).

Since there is a confound between age and age when obtained, because age is obviously dependent to some extent upon age when obtained, we repeated the analysis considering only cats aged more than 3 (Supplementary Tables 7–16) and the findings support our first aim, however there were differences in the relationships identified as part of our second aim. Fearfulness was excluded as a potential risk factor for both aggression and playfulness. There was also a change in the relationship between aggression of neutered vs. intact males: intact males were consistently more aggressive than neutered ones across the methods of calculation (Supplementary Table 11).

## Discussion

These results highlight the problems that can arise when behavioural survey data which conflate prevalence (i.e., which specific behaviours occur within a composite measure) with intensity (the frequency with which a particular behaviour occurs in a given context) is pooled to create a dependent variable for some form of further epidemiological investigation. We refer to this as a “pooling fallacy” as the result of bringing these two distinctive dimensions of a behavioural variable together into a single score is not a homogenous measure of the intensity/severity of the construct of interest. Our results show firstly how this can produce significant differences in the apparent factors of importance (age, breed type, scratching and spraying being differently significant depending on method used for calculation of aggression score and breed type, sex with neuter status, source of animal, lifestyle, time left alone, presence of dog, health status and scratching being variously significant when playfulness was calculated using five different methods), and, as predicted, how the greater the number of variables being pooled the greater the risk of error (four to 6 predictors being significant for aggression comprising of 4 items and 8–11 predictors being significant for playfulness factor comprising of 11 items). This is not surprising from a mathematical perspective as a larger number of variables mean there are a greater number of permutations that can give a given score. The problem is not unique to Fe-BARQ and C-BARQ and should be considered for any instrument that consists of items relating to constructs assessed for their severity in a range of contexts. There is no simple or single solution to this issue, other than to undertake multiple analyses using different cut off points as we have done here, and then consider carefully the results across analyses focusing on the most consistent effects. It is essential that researchers consider carefully the potential impact of such pooling and the limitation this might impose on their conclusions, and we encourage research to use multiple methods to evaluate the robustness of their results.

The “fallacy” in such pooling is assuming that the pooled score of a behavioural profile instrument is a reliable measure of the intensity of the behaviour within the individual, like the general level of a temperament trait. A behaviour is a response to a particular context, whereas a trait is a broader response tendency and so, by definition, should be evident across a range of contexts. Behavioural responses in a given context are often shaped by direct experience in that context. Thus, a behavioural profile can tell us about the animal's response in a range of particular circumstances, but this information relates only indirectly to the intensity of any underlying trait. For example, if a cat avoids a wide range of situations then we might conclude that it is temperamentally fearful, however if it avoids some situations but not others or has an extreme response in certain specific situations and not others, the temperament of the cat is much less clear. It is for this reason that Brady et al. (32) highlighted the need to develop a consensus on the difference between an instrument that produces a behavioural profile (which they refer to as an assessment of “character”), such as Fe-BARQ or C-BARQ (whose structure describes the aggregation of context specific behaviours), and one that assesses personality, such as the Monash Dog Personality Questionnaire (33, 34) or similar feline assessments (35–37) whose structure potentially describes broader biological based predispositions that underpin individual differences, and one that assesses temperament, such as the Positive and Negative Activation Scale (38) or Canine Frustration Questionnaire (39) whose structure is focused on the emotional aspects of personality. Each is valuable in its own right, and the appropriateness of one over another in a given context, will vary with the question being asked. We suggest that pooling the scores of a behavioural profile to suggest that we now have a measure of temperament is at best unreliable and at worst a fallacy. Thus, if we are interested in assessing the genetic basis to behavioural predispositions, we would suggest that the use of traits may provide greater accuracy than a factor based on behavioural profiles; this does not negate the feasibility of the latter approach [as recently demonstrated by (17)], but it may be less accurate than a more nuanced approach. However, if it is believed that there is a genetic basis to a contextually specific response then using a behavioural profile may be preferable. What is important is that we think carefully about what measure is best so that we can optimise our investigation, or at least recognise its limitations.

The variability in the results relating to different forms of average score, is not simply noise; it may be biologically meaningful and of practical value. For example, in the case of aggression score, the very high correlation between *Averaged total score* and *Averaged total binary score 1* indicates that for this factor the *Averaged total score* (which is the score that is most likely to be subject to a pooling fallacy) reflects the prevalence of this type of response in different contexts rather than its frequency within a given context. By contrast for playfulness, the strongest correlation was between *Averaged total score* and *Averaged total binary score 3*. This could indicate that *Averaged total score* for this trait reflects playfulness expressed very frequently (usually and always), however, it might also be an artefact of the nature of the distribution of scores. *Averaged total binary scores* for the two factors might therefore reflect different properties of the factor (prevalence at any level in the case of aggression, but the prevalence of frequent occurrences in the case of playfulness). This finding is reflected in the different frequency distribution histograms of aggression and playfulness scores (Supplementary Graphs 1–25) and an appreciation of the how average component scores are composed from constituent items is essential, if we wish to avoid unsound inferences.

It is also useful to consider what the variation in predictors with different cut off points might tell us. Again, these differences should not be simply dismissed as random errors, but their potential biological significance evaluated in terms of hypotheses that might need to be investigated. This is particularly valuable, when it is recognised that the work is exploratory [hypothesis generating research (12)] such as that described here. Indeed, we would argue that the primary goal of much initial epidemiological work examining the relationship between potential risk factors and outcomes should be to generate hypotheses, that can be more rigorously tested later on, and both the statistical plan and discussion of findings need to be considered accordingly (40). The exclusion of scratching as a potential risk factor only from the minimal adequate model for aggression relating to the *Averaged total binary score 1*, would suggest that inappropriate scratching, might be associated more with the frequency of bouts of aggression to familiar cats than the general prevalence or very low levels of the problem. Likewise, spraying was not a significant potential risk factor when only the data relating to the highest frequency of familiar cat aggression (always aggressive cats in different range of contexts) was used to calculate aggression score. This could suggest that spraying is associated less with the most severe cases of intercat aggression. Interestingly, there is some evidence to support this; within the clinical behavioural literature, it has been suggested that spraying is a response to low level of frustration associated with threats to resources to others, which may be the prelude to overt aggression (41). There are also data to indicate that pheromonal treatments which reliably moderate urine spraying in cats (42) are less effective when there is overt aggression between cats in the home (43). Both of these observations are consistent with the association identified in the current study and add weight to the hypothesis concerning the relationship between spraying and frustration.

The current analysis also reveals items that are consistently associated with a given factor: sex including neuter status, social play and fearfulness in the case of aggression scores and age, age when obtained, social play and fearfulness in the case of playfulness. This would suggest a very important role for these factors in these aspects of behaviour, even though some of the relationships are weak.

The relationship between sex/neuter status and aggressivity is clearly complex and has been frequently studied in cats, however, there seems to be little consensus on what the relationship is. Like many other species, male cats are often reported to be more aggressive than females (5, 44); however several studies have failed to find gender differences in aggression towards other cats (6, 8, 45, 46). This has led some to suggest that aggression in domestic cats lacks sexual dimorphism (8). Data from animal behaviour clinics, support our finding that females may be the more aggressive sex (47, 48), or at least the sex where it is perceived to be a problem. However, these clinical populations are often skewed towards neutered, mostly indoor house cats (5, 47–51), like those studied here. Unspecified generalisation about the relationship between sex and aggressivity, without considering factors such as neuter status, context (such as target of the behaviour) alongside the motivational-emotional status of the problem should therefore be treated with caution. Neutering has been reported to have a calming effect on behaviour with a decrease in aggression in both, males and females (44, 52); by contrast our initial results indicated aggressivity was consistently highest in female neutered cats, and greater in females compared to males. This might reflect the management of male entire cats, who are perhaps kept away from other cats to reduce the chances of unplanned breeding, but deserves further attention, as this would not account for the generally lower aggression in neutered males compared to females. Some clarification comes from our subsequent analysis which recognised the confound between age and age when obtained. In our initial analysis, entire males were less aggressive than neutered males, except for the most severe form of aggression (*Averaged total binary score 4*). This could be interpreted to indicate that neutering may have little effect on the general frequency of aggression in male cats, but it does reduce the most severe forms which could perhaps be related more to reproductive opportunities. In our subsequent analysis of cats 3 years old and more, intact males were consistently more aggressive than neutered ones, and not just in relation to ATBS 4 scores (see Supplementary Table 11). Thus, the relationship with neuter status in males, seems to be an artefact of many young entire males who are less aggressive. This might be revealed by a more sophisticated analysis which controls for such interactions, but this would hardly be justified in the exploratory phase. Nonetheless it does highlight the importance of using an incremental approach and series of analyses to build an understanding rather than depend on a single final analysis. It has been suggested that neutering may increase shyness and hiding in cats (53) and Ramos and Mills (8) reported an increased risk of human aggression when being startled by neutered cats. Other authors have also noted that neutering does not reduce fear induced aggression (54), and given our finding that fearfulness may increase aggressivity, this might provide a mechanism for the relationship found between neutering and aggression in both sexes.

The consistent relationship between aggressivity and both social play [Plays with other household cat(s)] and fearfulness [Runs and/or hides in response to sudden or loud noise (e.g., vacuum cleaner, car backfire, road drills, dropped object, sounds of musical instruments, doorbells or someone knocking on the door)], are as to be expected; as is the converse relationship between these two factors (aggression and fearfulness) and social play. However, these relationships, while being consistently significant do not appear as strong as might be predicted; this highlights the problem of simply relying on *p*-values for interpretation; an issue we discuss further below. This might also indicate that they are not strong determinants of aggressivity or object playfulness or be an artefact of the method used which depends heavily on owner interpretation for these items. Animals play less when their welfare is sub-optimal and this will include when they are fearful (20), so those who are afraid of more stimuli, can be expected to play less as a result; fearfulness is also a common underlying state that leads to aggression in animals (30). In particular, it has been reported that in cats that a fearful reaction to noises is most common triggering stimulus for redirected aggression (55). The strength of this relationship should be explored more rigorously through studies specifically designed to both test these hypotheses and quantify effect sizes. However, it must also be considered that these relationships are also affected by owner perception. Owners who see their cats fight more [especially if one is clearly fearful of another and thus likely to growl or hiss at each other (56)], may also tend to interpret rough and tumble play as aggression, which would conflate the reported relationship. However, our results indicate a negative relationship between social play and aggression, suggesting that, in general, this potential confusion was not the norm; however, it might weaken the relationship. Unfortunately, the definition of social play in Fe-BARQ and elsewhere is often defined using a circular definition (57): i.e., Plays with other household cat(s); without reference to more objective criteria such as inhibited biting and scratching which may be important ways of distinguishing it from affective aggression (58). Observational studies and specific hypothesis driven research to tease out these factors more clearly would be valuable.

The relationship between solitary/object play (items included with our playfulness factor) and social play with other household cats deserves further consideration. Panksepp (30) argues that the two have a different affective basis, and the weaker relationship here might support this. The original relationship between social and solitary play identified during the development of Fe-BARQ (3) may actually be an artefact of the use of a single item relating to social play. As a result, it may correlate more closely with items relating to solitary play, due to greater commonality between these items than the other states examined, however this does not mean they reflect a unitary “playful” system. If there had been more items relating to social play, it might be (if Panksepp's argument is correct) that these “social play” items would cross correlate more closely and thus form their own factor. This deserves further research attention, but an important prerequisite is the development of suitable items to use in the definition of social play. So far, there appears to have been only one observational analysis of intercat interactions involving both kittens and adult cats (59). This suggests that the patterning of behaviours such as wrestling, vocalisations and periods of inactivity might be useful for helping to differentiate between playful and agonistic encounters. The authors will be examining this further in their future research.

Age and age when obtained were both consistently associated with playfulness in the current study: playfulness declined with increasing age of the cat and also with increasing age when the animal was acquired for cats obtained at or after 2–6 months of age, with cats obtained before this slightly less playful too. Decline of playfulness with ageing is a common finding in developmental studies on play (60–62), and our result replicates that from another recent Fe-BARQ based study by Duffy et al. (3), which is important given the lack of developmental studies on cat play after the age of 6 months (57). This is a significant gap in the literature, which needs to be addressed. Burghardt (63) argues that play is initiated when an animal is well-fed and free from environmental stresses (e.g., physical danger, predators, social instability) or intense competing systems (e.g., feeding, mating, competition, fear), so the question arises how are these age-related factors related to freedom from environmental stressors and competing systems? It might be that when kittens are obtained very young (0–2 months of age), that their management is suboptimal in the absence of a mothering queen and this has knock on effects for their playfulness, whilst those obtained later than 6 months, may be more likely to have had a more traumatic early life, reflected in their later (re)homing. It should also be noted that between 2–6 months of age includes the developmental peak for play in cats (64). Accordingly, it might be that when cats are obtained at this age that there is greater reinforcement of the behaviour by owners through the provision and use of toys and interactive devices. While living in multicat households (such as in our population), provides the opportunity for social play (not the focus of playfulness in our study), it comes at a cost which includes potentially increased competition over resources and social conflict (23), and this may also contribute to the relationship between age when obtained and playfulness, as older cats may be faced with greater competition from resident animals, and less easily integrated. To date, studies of risk factors for intercat aggression (5, 6) do not appear to have considered this.

There are several limitations to this study which need to be put into context. Our primary aim was to illustrate how pooling scores that confound range of contexts with severity can result in different potential risk factors to a score based on specific threshold of response; our secondary aim was to explore and evaluate the potential risk factors identified by this process. The latter part of the report should be considered very much exploratory research aimed at generating hypotheses for future evaluation and it is this aim that is potentially affected by the main limitations of our chosen analyses. One concerns the statistical threshold used for considering significance and the other the lack of statistical correction for multiple testing. We have justified this decision earlier (40), but it should be noted any future research aimed at specifically testing the hypotheses we generate here should allow for such statistical correction. Secondly amongst the factors considered, we acknowledge that there are potential confounds, such as between age and age when obtained. The repeat the analysis considering only cats aged more than 3 did not affect our general aim, but did result in changes in specific factors of importance. We recognise that a more sophisticated analysis examining interactions between factors could more clearly reveal some of these points, but was not justified in this case, given our primary aim, which also emphasises the importance of an incremental approach to the analysis in exploratory research and not to treat the data as if they are suitable for hypothesis testing research. Although our work is exploratory, we have made reference to effect sizes and, it might be argued that this is not appropriate. Certainly, these measures should be treated with some caution until more definitive studies are undertaken, but they do provide some insight into what might be the most important/relevant factors to investigate in future. In line with a growing literature on the topic (65–68), we argue against a simple dependence of crude metrics and conventional threshold and wish to encourage a more thinking approach to analysis and reporting of this. Thirdly, number of cats per respondent filling the questionnaire was unknown in this study due to our anonymised data set, and while this fact does not invalidate the primary aim of our analysis, conclusion relating to potential risk factors may be influenced by interdependencies of the data from the same household.

In conclusion, failure to recognise the “pooling fallacy” of creating a heterogeneous total score for a dependent variable can result in different predictors appearing to be statistically significant. It is essential that researchers reflect carefully on what pooling scores actually produces and the implications of this. We argue that by converting component item scores into binary outcomes based on a particular threshold, a richness to the data may be revealed that could otherwise be lost. There is clearly a need for researchers to develop scales which do not suffer from this problem. Further we urge researchers to consider carefully whether their research is aimed at generating tentative hypotheses for subsequently evaluation or aimed at providing definitive evidence concerning a specific hypothesis and to adapt their analyses and reporting accordingly.

## Data Availability Statement

The raw data supporting the conclusions of this article will be made available by the authors, without undue reservation.

## Ethics Statement

Ethical review and approval was not required for the animal study because the experimental protocol used in this study (internet questionnaire survey) did not require approval by the local Ethics committee of the University of Veterinary Medicine and Pharmacy in Košice for handling of animals, as this ethics committee was established by University of Veterinary Medicine and Pharmacy in Košice based on Decree of Ministry of Agriculture and Rural Development of the Slovak Republic laying down details on requirements for the protection of animals used for scientific and educational purposes (Decree No. 436/2012 coll.) and following these regulations, experimental protocols/projects where animals are used indirectly, *via* questionnaire assessment filled by their owners, do not require approval of Ethics committee of the University of Veterinary Medicine and Pharmacy in Košice for handling of animals. Thus, all experiments within this study (the questionnaire survey and its analysis) were performed in accordance with guidelines and regulations of University of Veterinary medicine and Pharmacy in Košice. Written informed consent for participation was not obtained from the owners because the online completed survey used in this study was directed at owners and it was assumed that all participants were over 18 and participants' consent was inferred from the completion of the survey and submission of the data. No sensitive information was gathered from any of the participants and any incomplete surveys, which might indicate withdrawal of consent, were not used.

## Author Contributions

NGK and DM contributed to the conception and design of the work and contributed to the analysis and interpretation of the data. RW collected the data. NGK, BP, JK, and DM drafted and revised the manuscript. All authors contributed to manuscript revision and approved submitted version.

## Conflict of Interest

The authors declare that the research was conducted in the absence of any commercial or financial relationships that could be construed as a potential conflict of interest.
