# Supplementary material for: Potential Risk Factors for Aggression and Playfulness in Cats: Examination of a Pooling Fallacy Using Fe-BARQ as an Example
Source: Front Vet Sci. 2021 Jan 5;7:545326. doi: 10.3389/fvets.2020.545326 (PMC7813754; doi:10.3389/fvets.2020.545326)
Supplement: Supplementary file 1 [file Data_Sheet_1.docx]

Supplementary Material - Questionnaire

Feline Behavioural Assessment & Research Questionnaire - FeBARQ

*Feline Behavioural Assessment & Research Questionnaire (FeBARQ)^©^2017 James A. Serpell*

The FeBARQ is designed to provide cat owners and professionals with standardised evaluations of feline temperament and behaviour.

Note: The FeBARQ is not designed to assess the behaviour of cats less than six months of age. If your cat is not yet six months old, we recommend adding him or her for assessment at a later time.

## Email address

1. **Postcode**
2. **Country**

*Item could have been answered by using drop-down list of countries.*

## Would you be interested in participating in future questionnaires regarding your cat (including making videos showing certain behaviours)?

Yes No

**Cat's Information**

## Cat's Name

1. **Age (years):**

6 months - 1 year

1

2

3

4

5

6

7

8

9

10

Over 11

## Breed type:

Domestic short hair/mixed breed Domestic long hair/mixed breed Pure breed (Pedigree)

Mix of pure breeds (or if only one parent of your cat is pedigree)

## Breed (check up to three boxes, if cat is a mix of known breeds). Leave blank if moggie / domestic /no known breed:

*Item could have been answered by using drop-down list of breeds.*

**5. Sex**

Male Female

## 6. Weight (approximate):

Less than 1 kg 1 - 3 kg

3 - 5 kg

5 - 8 kg

8 - 11 kg

Over 11 kg

## 7. Obtained from:

Home (born at) Friend/relative/ neighbour Veterinary hospital Street (as a stray) Shelter

Breeding cattery

Cattery (boarding/foster) Pet store (purchase) Pet store (rescue) Selling website

## Age when obtained:

Unweaned kitten (0 - 2 months)

Kitten (2 - 6 months)

Junior (6 months - 2 years) Mature (3 - 10years) Senior (11+ years)

# Health & Lifestyle

## Is the cat neutered? (spayed/castrated):

Yes No

## Age when neutered (leave blank if unknown):

Under 3 months

3 - 6 months

6 months - 1 year

over 2 years

## Primary reason for neutering:

To control reproduction

To prevent or reduce aggression

To prevent or control urine marking, to reduce odour from urine

To prevent unwanted behaviour associated with mating season (vocalisation, wandering...)

Not known Other

1. **Home environment:**

Flat HouseFarm

Cattery (boarding/foster) Breeding cattery

Shelter

## Lifestyle:

Indoors only

Indoors with controlled or limited access to space outdoors (e.g. fenced garden or pen) Indoors with free access outdoors

Outside only (no access indoors)

Lives in pen/stall/cage and has controlled access outdoors

## Where does cat usually sleep at night?

In bedroom on bed (with a household member) In bedroom but not on bed

In another room in house

Caged or confined in part of house Caged or confined in outdoor run/shed Outside free-ranging

## In a typical week, how many days is this cat left alone (no people around) at home for more than 1 hour?

Zero 1 - 3

3 - 5

5 - 7

## On a typical day, how many hours is this cat left alone (no people around) at home?

0 - 1

1 - 2

2 - 4

4 - 6

6 - 8

8 - 12

13 or more

## Is this cat currently suffering from any health problems?

No Yes

## If yes, please describe briefly (10 words or less):

1. **Are you currently experiencing any problems with this cat’s behaviour or temperament?**

No

Only minor problems Moderate problems Serious problems

## If so, please describe briefly (10 words or less):

**Ownership Information**

1. **What is your primary reason for owning this cat?**

As a pet/companion Rodent control

Breeding and/or showing

Other

## If you selected "Other" please briefly describe your reason for owning this cat:

1. **Is this the first cat you have ever owned?**

Yes No

## If no, how many have you owned before?

1 - 2

3 - 5

6 - 10

10 or more

## Did you grow up with cats as a child (from 0 - 16 years)?

Yes No

## How many cats are kept in your household all together?

Only 1

2 - 5

5 - 10

10 or more

## Does a dog live in your household?

Yes No

# General Activity/Playfulness

Some cats are more active and playful than others. Please indicate how playful and active your cat has been recently in each of the following contexts. Check the "unknown" column if you have never observed the cat in the situation described.

# Section 1: Activity/Playfulness

## Quickly learns how to play with new introduced toys.

0 = Never 1 = Seldom 2 = Sometimes 3= Usually 4 = Always (If Unknown, leave question blank)

0 1 2 3 4

Never Always

## Curious: actively investigates/explores new objects, sights, or changes in its environment.

0 = Never 1 = Seldom 2 = Sometimes 3= Usually 4 = Always (If Unknown, leave question blank)

0 1 2 3 4

Never Always

## Carries small objects/toys in the mouth to interact with.

0 = Never 1 = Seldom 2 = Sometimes 3= Usually 4 = Always (If Unknown, leave question blank)

0 1 2 3 4

Never Always

## Runs and jumps in the air.

0 = Never 1 = Seldom 2 = Sometimes 3= Usually 4 = Always (If Unknown, leave question blank)

0 1 2 3 4

Never Always

## Engages in active jumping and climbing on high surfaces, furniture or curtains.

0 = Never 1 = Seldom 2 = Sometimes 3= Usually 4 = Always (If Unknown, leave question blank)

0 1 2 3 4

Never Always

## Exhibits sudden bursts of running or climbing in certain periods of the day.

0 = Never 1 = Seldom 2 = Sometimes 3= Usually 4 = Always (If Unknown, leave question blank)

0 1 2 3 4

Never Always

## Exhibits sudden jumping and running during playful activity.

0 = Never 1 = Seldom 2 = Sometimes 3= Usually 4 = Always (If Unknown, leave question blank)

0 1 2 3 4

Never Always

1. **Stalks, chases or pounces on moving objects (string, balls, soft toys, etc.) during playful activity.**

0 = Never 1 = Seldom 2 = Sometimes 3= Usually 4 = Always (If Unknown, leave question blank)

0 1 2 3 4

Never Always

## Displays running/chasing and hunting/pouncing on unseen/imaginary prey/objects.

0 = Never 1 = Seldom 2 = Sometimes 3= Usually 4 = Always (If Unknown, leave question blank)

0 1 2 3 4

Never Always

## Chases and ambushes other household members (including pets) playfully.

0 = Never 1 = Seldom 2 = Sometimes 3= Usually 4 = Always (If Unknown, leave question blank)

0 1 2 3 4

Never Always

## Chases or follows shadows or light spots.

0 = Never 1 = Seldom 2 = Sometimes 3= Usually 4 = Always (If Unknown, leave question blank)

0 1 2 3 4

Never Always

## Initiates mutual chasing by running from room to room in the house.

0 = Never 1 = Seldom 2 = Sometimes 3= Usually 4 = Always (If Unknown, leave question blank)

0 1 2 3 4

Never Always

## Initiates interactive play with people in the home (i.e. brings toys, strings, or small objects to play with).

0 = Never 1 = Seldom 2 = Sometimes 3= Usually 4 = Always (If Unknown, leave question blank)

0 1 2 3 4

Never Always

## Plays with other household cat(s) - leave question blank if this is the only cat in the

## household.

0 = Never 1 = Seldom 2 = Sometimes 3= Usually 4 = Always (If Unknown, leave question blank)

0 1 2 3 4

Never Always

# Sociability

Some cats are more sociable and friendly than others. Please indicate how sociable your cat has been recently in each of the following circumstances. Check the "unknown" column, if you have never observed the cat in the situation described.

# Section 2: Sociability with people

## Is comfortable and relaxed among people in social gatherings.

0 = Never 1 = Seldom 2 = Sometimes 3= Usually 4 = Always (If Unknown, leave question blank)

0 1 2 3 4

Never Always

## Is comfortable and relaxed being petted by unfamiliar (non- household) person(s).

0 = Never 1 = Seldom 2 = Sometimes 3= Usually 4 = Always (If Unknown, leave question blank)

0 1 2 3 4

Never Always

## Greets unfamiliar adults visiting your home in a friendly manner (sniffs, rubs, purrs, lies on the floor).

0 = Never 1 = Seldom 2 = Sometimes 3= Usually 4 = Always (If Unknown, leave question blank)

0 1 2 3 4

Never Always

## Greets unfamiliar children visiting your home in a friendly manner (sniffs, rubs, purrs, lies on the floor).

0 = Never 1 = Seldom 2 = Sometimes 3= Usually 4 = Always (If Unknown, leave question blank)

0 1 2 3 4

Never Always

1. **Appears comfortable (confident, relaxed) when playing with familiar child(ren).**

0 = Never 1 = Seldom 2 = Sometimes 3= Usually 4 = Always (If Unknown, leave question blank)

0 1 2 3 4

Never Always

## Appears comfortable (confident, relaxed) when playing with unfamiliar child(ren).

0 = Never 1 = Seldom 2 = Sometimes 3= Usually 4 = Always (If Unknown, leave question blank)

0 1 2 3 4

Never Always

## Appears comfortable (confident, relaxed) when playing with unfamiliar adults.

0 = Never 1 = Seldom 2 = Sometimes 3= Usually 4 = Always (If Unknown, leave question blank)

0 1 2 3 4

Never Always

# Section 3: Directed calls/vocalisations

## "Talks" to people using calls/vocalisations (i.e. answers when spoken to by a person).

0 = Never 1 = Seldom 2 = Sometimes 3= Usually 4 = Always (If Unknown, leave question blank)

0 1 2 3 4

Never Always

## Asks (vocalises, walks toward door, makes noise) to be let outside, or in and out of rooms.

0 = Never 1 = Seldom 2 = Sometimes 3= Usually 4 = Always (If Unknown, leave question blank)

0 1 2 3 4

Never Always

## Asks (vocalises, walks toward its bowl or food source, makes noise) for food when hungry.

0 = Never 1 = Seldom 2 = Sometimes 3= Usually 4 = Always (If Unknown, leave question blank)

0 1 2 3 4

Never Always

1. **Meows loudly in front of any closed door.**

0 = Never 1 = Seldom 2 = Sometimes 3= Usually 4 = Always (If Unknown, leave question blank)

0 1 2 3 4

Never Always

# Section 4: Purring

## Purrs when stroked or petted.

0 = Never 1 = Seldom 2 = Sometimes 3= Usually 4 = Always (If Unknown, leave question blank)

0 1 2 3 4

Never Always

## Purrs when sitting/lying on someone’s lap.

0 = Never 1 = Seldom 2 = Sometimes 3= Usually 4 = Always (If Unknown, leave question blank)

0 1 2 3 4

Never Always

# Section 5: Attention seeking

## Nudges and/or nuzzles you or other members of the household when you are sitting or lying down.

0 = Never 1 = Seldom 2 = Sometimes 3= Usually 4 = Always (If Unknown, leave question blank)

0 1 2 3 4

Never Always

## Seeks out physical contact with you or other household members when you/they are sitting or lying down.

0 = Never 1 = Seldom 2 = Sometimes 3= Usually 4 = Always (If Unknown, leave question blank)

0 1 2 3 4

Never Always

# Section 6: Sociability with cats

## Greets unfamiliar (non- household) cats visiting your home in a friendly manner (sniffs, touches nose, rubs).

0 = Never 1 = Seldom 2 = Sometimes 3= Usually 4 = Always (If Unknown, leave question blank)

0 1 2 3 4

Never Always

## Approaches unfamiliar adult cats outside your home in a friendly manner (sniffs, touches noses, rubs).

0 = Never 1 = Seldom 2 = Sometimes 3= Usually 4 = Always (If Unknown, leave question blank)

0 1 2 3 4

Never Always

## Approaches unfamiliar kittens outside your home in a friendly manner (sniffs, touches noses, rubs).

0 = Never 1 = Seldom 2 = Sometimes 3= Usually 4 = Always (If Unknown, leave question blank)

0 1 2 3 4

Never Always

# Antisocial or Aggressive Behaviour

Some cats exhibit threatening or hostile behaviour from time to time or in particular situations. Please indicate how often your cat has shown threats or hostility in each the following contexts. Check the "unknown" column, if you have never observed the cat in the situation described

# Section 7: Stranger-directed aggression

## Growls/hisses when an unfamiliar (non- household) person tries to touch or pet him/her.

0 = Never 1 = Seldom 2 = Sometimes 3= Usually 4 = Always (If Unknown, leave question blank)

0 1 2 3 4

Never Always

## Growls/hisses at unfamiliar child(ren) visiting its home.

0 = Never 1 = Seldom 2 = Sometimes 3= Usually 4 = Always (If Unknown, leave question blank)

0 1 2 3 4

Never Always

1. **Growls/hisses at unfamiliar adult(s) visiting its home.**

0 = Never 1 = Seldom 2 = Sometimes 3= Usually 4 = Always (If Unknown, leave question blank)

0 1 2 3 4

Never Always

# Section 8: Touch sensitivity/Owner-directed aggression

## Scratches/bites or attempts to bite (in a non-playful way) when petted on the belly.

0 = Never 1 = Seldom 2 = Sometimes 3= Usually 4 = Always (If Unknown, leave question blank)

0 1 2 3 4

Never Always

## Growls, hisses, scratches or bites when stroked along the back or spine.

0 = Never 1 = Seldom 2 = Sometimes 3= Usually 4 = Always (If Unknown, leave question blank)

0 1 2 3 4

Never Always

## Lashes out (scratches, bites) unexpectedly when petted.

0 = Never 1 = Seldom 2 = Sometimes 3= Usually 4 = Always (If Unknown, leave question blank)

0 1 2 3 4

Never Always

## Chases, grabs onto, or attacks people's legs or feet in movement (in a non-playful way).

0 = Never 1 = Seldom 2 = Sometimes 3= Usually 4 = Always (If Unknown, leave question blank)

0 1 2 3 4

Never Always

# Section 9: Resistance to Restraint

## Growls, hisses, scratches or bites when given medicine by a familiar person.

0 = Never 1 = Seldom 2 = Sometimes 3= Usually 4 = Always (If Unknown, leave question blank)

0 1 2 3 4

Never Always

## Growls, hisses, scratches or bites when being bathed.

0 = Never 1 = Seldom 2 = Sometimes 3= Usually 4 = Always (If Unknown, leave question blank)

0 1 2 3 4

Never Always

## Growls, hisses, scratches or bites when being groomed.

0 = Never 1 = Seldom 2 = Sometimes 3= Usually 4 = Always (If Unknown, leave question blank)

0 1 2 3 4

Never Always

## Growls, hisses, scratches or bites when nails/claws are clipped.

0 = Never 1 = Seldom 2 = Sometimes 3= Usually 4 = Always (If Unknown, leave question blank)

0 1 2 3 4

Never Always

# Section 10: Familiar cat aggression (if your cat is the only cat in the household, please leave these questions blank - Q44, 45,

**46, 47).**

## Growls/hisses when approached by a familiar (household) cat while eating.

0 = Never 1 = Seldom 2 = Sometimes 3= Usually 4 = Always (If Unknown, leave question blank)

0 1 2 3 4

Never Always

## Growls/hisses when approached by a familiar (household) cat at a favourite resting place.

0 = Never 1 = Seldom 2 = Sometimes 3= Usually 4 = Always (If Unknown, leave question blank)

0 1 2 3 4

Never Always

## Growls/hisses when stared at, growled or hissed at by a familiar (household) cat.

0 = Never 1 = Seldom 2 = Sometimes 3= Usually 4 = Always (If Unknown, leave question blank)

0 1 2 3 4

Never Always

## Attacks (scratches/bites/attempts to bite) when stared at, growled or hissed at by a familiar (household) cat.

0 = Never 1 = Seldom 2 = Sometimes 3= Usually 4 = Always (If Unknown, leave question blank)

0 1 2 3 4

Never Always

# Section 11: Dog Aggression (if this is an indoor cat who does not live with a dog, you may wish to leave some of these

**questions blank).**

## Growls/hisses at familiar dog(s).

0 = Never 1 = Seldom 2 = Sometimes 3= Usually 4 = Always (If Unknown, leave question blank)

0 1 2 3 4

Never Always

## Attacks (scratches/bites/attempts to bite) familiar dog(s).

0 = Never 1 = Seldom 2 = Sometimes 3= Usually 4 = Always (If Unknown, leave question blank)

0 1 2 3 4

Never Always

## Growls/hisses when unfamiliar dog visits its home or enters its yard/garden.

0 = Never 1 = Seldom 2 = Sometimes 3= Usually 4 = Always (If Unknown, leave question blank)

0 1 2 3 4

Never Always

## Attacks (scratches/bites/attempts to bite) when unfamiliar dog visits its home or enters its yard/garden.

0 = Never 1 = Seldom 2 = Sometimes 3= Usually 4 = Always (If Unknown, leave question blank)

0 1 2 3 4

Never Always

## Chases or scratches unfamiliar dogs on the street, if given the opportunity.

0 = Never 1 = Seldom 2 = Sometimes 3= Usually 4 = Always (If Unknown, leave question blank)

0 1 2 3 4

Never Always

# Shyness or Nervousness

Some cats are insecure or frightened in certain situations. Please indicate how often your cat has shown signs of shyness or nervousness in each of the situations described below. Check the "unknown" column, if you have never observed the cat in the situation described.

# Section 12: Fear of unfamiliar dogs/cats

## Runs and/or hides when unfamiliar (non- household) cats visit its home or enter its yard/garden.

0 = Never 1 = Seldom 2 = Sometimes 3= Usually 4 = Always (If Unknown, leave question blank)

0 1 2 3 4

Never Always

## Runs and/or hides when unfamiliar dogs visit its home or enter its yard/garden.

0 = Never 1 = Seldom 2 = Sometimes 3= Usually 4 = Always (If Unknown, leave question blank)

0 1 2 3 4

Never Always

# Section 13: Fear of novelty

## Shows restlessness (active investigation) when its resting area is modified (e.g. objects moved from usual place, changing fabrics/sheets, etc).

0 = Never 1 = Seldom 2 = Sometimes 3= Usually 4 = Always (If Unknown, leave question blank)

0 1 2 3 4

Never Always

## Shows restlessness (active investigation) or hyper vigilance (constant ear movements and watchfulness) when unfamiliar objects are introduced into the home.

0 = Never 1 = Seldom 2 = Sometimes 3= Usually 4 = Always (If Unknown, leave question blank)

0 1 2 3 4

Never Always

# Attachment and Separation

Some cats show signs of anxiety or abnormal behaviour when separated from their owners. Thinking back over the recent past, how often has your cat shown each of the following signs of separation- related behaviour when left, or about to be left, on its own. Check the "unknown" column, if you have never observed this cat in the situation described.

# Section 14: Separation-related behaviour

## Shows restlessness, agitation and/or pacing when you or another household member prepares to leave the home.

0 = Never 1 = Seldom 2 = Sometimes 3= Usually 4 = Always (If Unknown, leave question blank)

0 1 2 3 4

Never Always

## Sulks, hides and/or slinks away when you or another household member prepares to leave the home.

0 = Never 1 = Seldom 2 = Sometimes 3= Usually 4 = Always (If Unknown, leave question blank)

0 1 2 3 4

Never Always

## Lies down or stays still in the vicinity of the entrance door when you or another household member prepares to leave the home.

0 = Never 1 = Seldom 2 = Sometimes 3= Usually 4 = Always (If Unknown, leave question blank)

0 1 2 3 4

Never Always

## Displays restlessness (active investigation) when left alone at home.

0 = Never 1 = Seldom 2 = Sometimes 3= Usually 4 = Always (If Unknown, leave question blank)

0 1 2 3 4

Never Always

## Remains still and alert/hyper vigilant (constant ear movements and watchful eyes) when left alone at home.

0 = Never 1 = Seldom 2 = Sometimes 3= Usually 4 = Always (If Unknown, leave question blank)

0 1 2 3 4

Never Always

## Vocalises by crying or meowing when left alone at home.

0 = Never 1 = Seldom 2 = Sometimes 3= Usually 4 = Always (If Unknown, leave question blank)

0 1 2 3 4

Never Always

# Other Behaviour

Cats display a wide range of behaviours in addition to those already covered by this questionnaire. Please indicate how often your cat has shown any of the following behaviours. Check the "unknown" column, if you have never observed the cat in the situation described.

# Section 15: Trainability

## Comes when called.

0 = Never 1 = Seldom 2 = Sometimes 3= Usually 4 = Always (If Unknown, leave question blank)

0 1 2 3 4

Never Always

## Readily responds to simple commands (out, in, quiet, down, up, no, lie down, etc).

0 = Never 1 = Seldom 2 = Sometimes 3= Usually 4 = Always (If Unknown, leave question blank)

0 1 2 3 4

Never Always

## Attends and listens closely to everything you say or do.

0 = Never 1 = Seldom 2 = Sometimes 3= Usually 4 = Always (If Unknown, leave question blank)

0 1 2 3 4

Never Always

# Section 16: Predatory Behaviour

## Brings prey animals (rodents, birds, reptiles, frogs, insects, worms, etc) into the home, given the opportunity.

0 = Never 1 = Seldom 2 = Sometimes 3= Usually 4 = Always (If Unknown, leave question blank)

0 1 2 3 4

Never Always

## Chases rodents, birds, reptiles, squirrels, rabbits, or other small animals, given the opportunity.

0 = Never 1 = Seldom 2 = Sometimes 3= Usually 4 = Always (If Unknown, leave question blank)

0 1 2 3 4

Never Always

## Is fascinated by the activities of other small pets (rodents, birds, reptiles, fish, etc) in the home.

0 = Never 1 = Seldom 2 = Sometimes 3= Usually 4 = Always (If Unknown, leave question blank).

0 1 2 3 4

Never Always

# Section 17: Prey interest

## Makes chirping or chattering noises when observing birds or other small animals outside the home.

0 = Never 1 = Seldom 2 = Sometimes 3= Usually 4 = Always (If Unknown, leave question blank)

0 1 2 3 4

Never Always

## Displays lashing tail, skin rippling and/or a tense body when looking at birds or other movements through the window.

0 = Never 1 = Seldom 2 = Sometimes 3= Usually 4 = Always (If Unknown, leave question blank)

0 1 2 3 4

Never Always

# Section 18: Location preferences for resting/sleeping

## Tends to rest/sleep in elevated places (shelves, bookcases, tops of wardrobes or cupboards, etc).

0 = Never 1 = Seldom 2 = Sometimes 3= Usually 4 = Always (If Unknown, leave question blank)*.*

0 1 2 3 4

Never Always

## Tends to sleep/rest on top of warm appliances (DVD player, TV, printer, computer, radiator, etc).

0 = Never 1 = Seldom 2 = Sometimes 3= Usually 4 = Always (If Unknown, leave question blank)

0 1 2 3 4

Never Always

## Tends to sleep/rest inside cupboards, clothes drawers, laundry baskets, etc.

0 = Never 1 = Seldom 2 = Sometimes 3= Usually 4 = Always (If Unknown, leave question blank)

0 1 2 3 4

Never Always

# Section 19: Excessive/compulsive self grooming

## Shows excessive and intensive grooming (e.g. several times a day for long periods).

0 = Never 1 = Seldom 2 = Sometimes 3= Usually 4 = Always (If Unknown, leave question blank)

0 1 2 3 4

Never Always

## Exhibits self-mutilation, hair barbering (pulls fur with teeth, vigorously nibbles and/or licks its body parts).

0 = Never 1 = Seldom 2 = Sometimes 3= Usually 4 = Always (If Unknown, leave question blank)

0 1 2 3 4

Never Always

## Exhibits sudden frantic licking or chewing.

0 = Never 1 = Seldom 2 = Sometimes 3= Usually 4 = Always (If Unknown, leave question blank)

0 1 2 3 4

Never Always

# Section 20: Other compulsive behaviours

## Stares intently at people.

0 = Never 1 = Seldom 2 = Sometimes 3= Usually 4 = Always (If Unknown, leave question blank)

0 1 2 3 4

Never Always

## Freezes and stares intently at nothing visible.

0 = Never 1 = Seldom 2 = Sometimes 3= Usually 4 = Always (If Unknown, leave question blank)

0 1 2 3 4

Never Always

## Shows strange repetitive movements (pacing, walking) from one place to another (short distances).

0 = Never 1 = Seldom 2 = Sometimes 3= Usually 4 = Always (If Unknown, leave question blank)

0 1 2 3 4

Never Always

# Section 21: Inappropriate elimination

## Urinates (crouching position) outside of the litter box or in other inappropriate areas indoors.

0 = Never 1 = Seldom 2 = Sometimes 3= Usually 4 = Always (If Unknown, leave question blank)

0 1 2 3 4

Never Always

## Defecates outside of the litter box or in other inappropriate areas indoors.

0 = Never 1 = Seldom 2 = Sometimes 3= Usually 4 = Always (If Unknown, leave question blank)

0 1 2 3 4

Never Always

# Section 22: Elimination preferences

## Shows location preferences for toileting (e.g. quiet, high or hidden places).

0 = Never 1 = Seldom 2 = Sometimes 3= Usually 4 = Always (If Unknown, leave question blank)

0 1 2 3 4

Never Always

## Shows substrate preferences for toileting (e.g., litter type, sand pebbles, grass, newspaper, bedding, laundry).

0 = Never 1 = Seldom 2 = Sometimes 3= Usually 4 = Always (If Unknown, leave question blank)

0 1 2 3 4

Never Always

# Section 23: Crepuscular activity

## Shows increased activity in the evening around dusk/sunset or late at night.

0 = Never 1 = Seldom 2 = Sometimes 3= Usually 4 = Always (If Unknown, leave question blank)

0 1 2 3 4

Never Always

## Shows increased activity in the early hours of morning around dawn/sunrise; maywake members of the household early in the morning.

0 = Never 1 = Seldom 2 = Sometimes 3= Usually 4 = Always (If Unknown, leave question blank)

0 1 2 3 4

Never Always

# Section 24: Miscellaneous behaviours

## Shows agitation, restlessness, or vocalisation when you or another member of the household shows affection for another person, cat or animal.

0 = Never 1 = Seldom 2 = Sometimes 3= Usually 4 = Always (If Unknown, leave question blank)

0 1 2 3 4

Never Always

## Gives sudden and loud vocalisations (meowing, yowling) either during the daytime or at night (without another cat or animal in sight).

0 = Never 1 = Seldom 2 = Sometimes 3= Usually 4 = Always (If Unknown, leave question blank)

0 1 2 3 4

Never Always

## Sprays (standing position with tail raised vertically) outside of the litter box or on other surfaces and objects (e.g., furniture, walls, people's legs, etc.) indoors.

0 = Never 1 = Seldom 2 = Sometimes 3= Usually 4 = Always (If Unknown, leave question blank)

0 1 2 3 4

Never Always

## Appears uncomfortable (trembles, becomes rigid/tense, struggles) when picked up/held in arms and/or when sitting on laps.

0 = Never 1 = Seldom 2 = Sometimes 3= Usually 4 = Always (If Unknown, leave question blank)

0 1 2 3 4

Never Always

## Growls, hisses, scratches or bites when approached while in possession of stolen food or prey.

0 = Never 1 = Seldom 2 = Sometimes 3= Usually 4 = Always (If Unknown, leave question blank)

0 1 2 3 4

Never Always

1. **Plays ‘fetch’; likes to retrieve thrown objects or toys.**

0 = Never 1 = Seldom 2 = Sometimes 3= Usually 4 = Always (If Unknown, leave question blank)

0 1 2 3 4

Never Always

## Readily adapts to changes in daily routines (schedules, replacement or rearrangement of objects and furniture).

0 = Never 1 = Seldom 2 = Sometimes 3= Usually 4 = Always (If Unknown, leave question blank)

0 1 2 3 4

Never Always

## Growls, hisses, scratches or bites when examined or treated by a veterinarian.

0 = Never 1 = Seldom 2 = Sometimes 3= Usually 4 = Always (If Unknown, leave question blank)

0 1 2 3 4

Never Always

## Runs and/or hides in response to sudden or loud noise (e.g. vacuum cleaner, car backfire, road drills, dropped object, sounds of musical instruments, doorbells or someone knocking on the door).

0 = Never 1 = Seldom 2 = Sometimes 3= Usually 4 = Always (If Unknown, leave question blank)

0 1 2 3 4

Never Always

## Escapes or attempts to escape from the home or yard/garden, if given the opportunity.

0 = Never 1 = Seldom 2 = Sometimes 3= Usually 4 = Always (If Unknown, leave question blank)

0 1 2 3 4

Never Always

## Scratches claws on inappropriate objects or surfaces indoors (furniture, rugs, curtains, wallpaper, etc.).

0 = Never 1 = Seldom 2 = Sometimes 3= Usually 4 = Always (If Unknown, leave question blank)

0 1 2 3 4

Never Always

1. **Tends to lie on paper, books or things that are being used by people.**

0 = Never 1 = Seldom 2 = Sometimes 3= Usually 4 = Always (If Unknown, leave question blank)

0 1 2 3 4

Never Always

## Chases its own tail/hind end.

0 = Never 1 = Seldom 2 = Sometimes 3= Usually 4 = Always (If Unknown, leave question blank)

0 1 2 3 4

Never Always

## Kneads, suckles, chews and/or mounts soft toys, fabrics, pillows, etc.

0 = Never 1 = Seldom 2 = Sometimes 3= Usually 4 = Always (If Unknown, leave question blank)

0 1 2 3 4

Never Always

## Chews or damages inappropriate objects when left alone at home (destructive behaviour).

0 = Never 1 = Seldom 2 = Sometimes 3= Usually 4 = Always (If Unknown, leave question blank)

*Mark only one oval.*

0 1 2 3 4

Never Always
