# Supplementary material for: Potential Risk Factors for Aggression and Playfulness in Cats: Examination of a Pooling Fallacy Using Fe-BARQ as an Example
Source: Front Vet Sci. 2021 Jan 5;7:545326. doi: 10.3389/fvets.2020.545326 (PMC7813754; doi:10.3389/fvets.2020.545326)
Supplement: Supplementary file 2 [file Data_Sheet_2.docx]

Supplementary Material

# Supplementary graphs

##
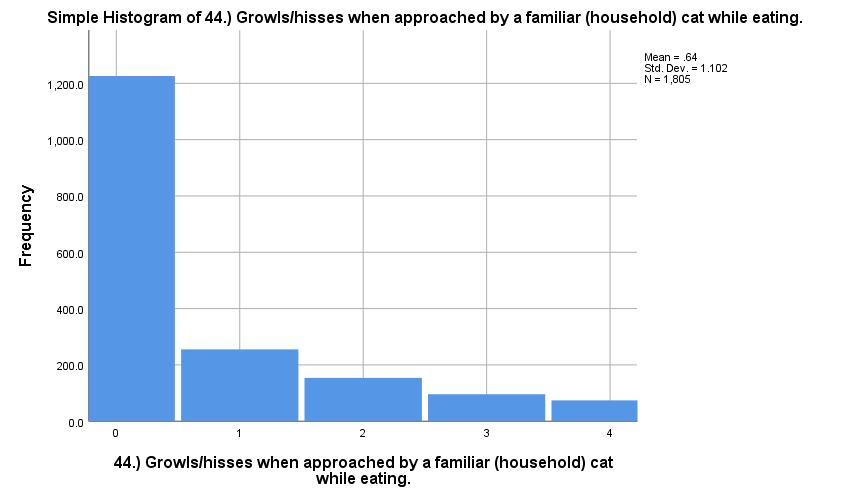
Supplementary Graph 1Frequency distribution histogram for aggression item 44.) Growls/hisses when approached by a familiar (household) cat while eating, x axis- frequency of behaviour occurrence: 0=never, 1=seldom, 2=sometimes, 3=usually, 4=always


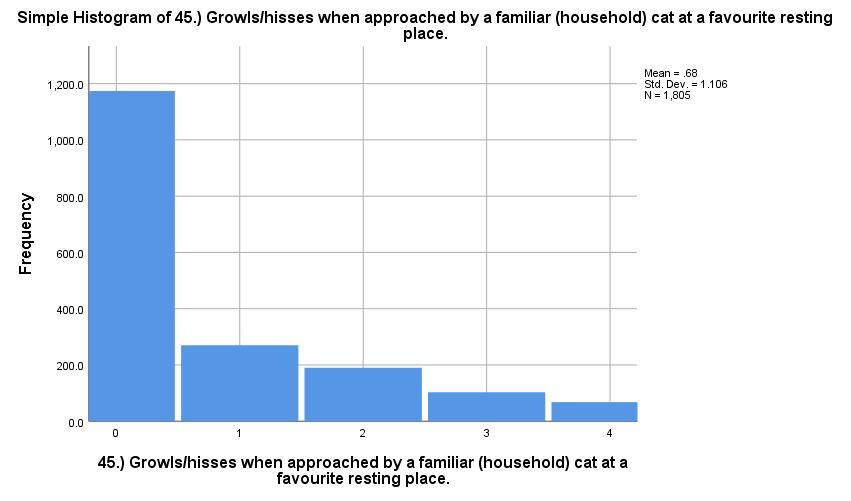


Supplementary Graph 2 Frequency distribution histogram for aggression item 45.) Growls/hisses when approached by a familiar (household) cat at a favourite resting place, x axis- frequency of behaviour occurrence:0=never, 1=seldom, 2=sometimes, 3=usually, 4=always


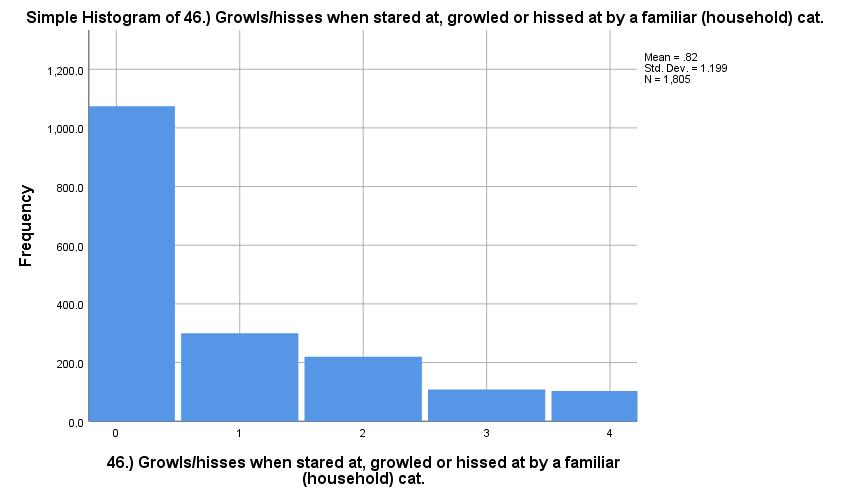


Supplementary Graph 3 Frequency distribution histogram for aggression item 46.) Growls/hisses when stared at, growled or hissed at by a familiar (household) cat, x axis- frequency of behaviour occurrence: 0=never, 1=seldom, 2=sometimes, 3=usually, 4=always


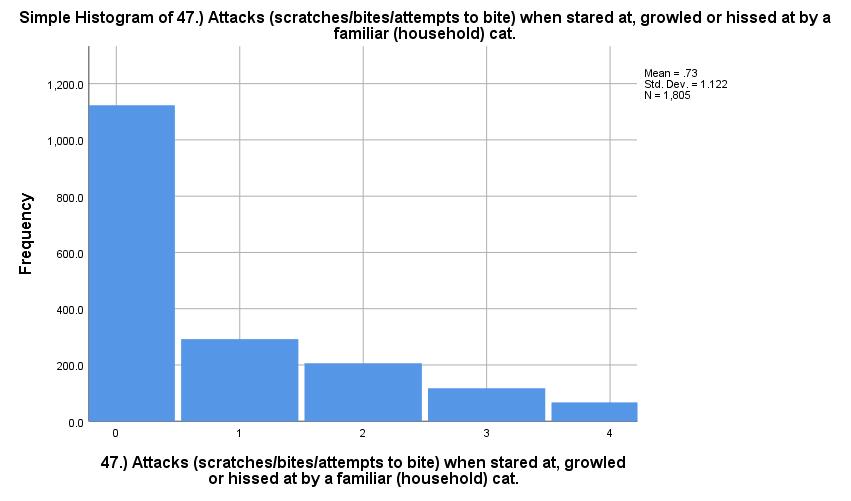


Supplementary Graph 4 Frequency distribution histogram for aggression item 47.) Attacks (scratches/bites/attempts to bite) when stared at, growled or hissed at by a familiar (household) cat, x axis- frequency of behaviour occurrence:0=never, 1=seldom, 2=sometimes, 3=usually, 4=always


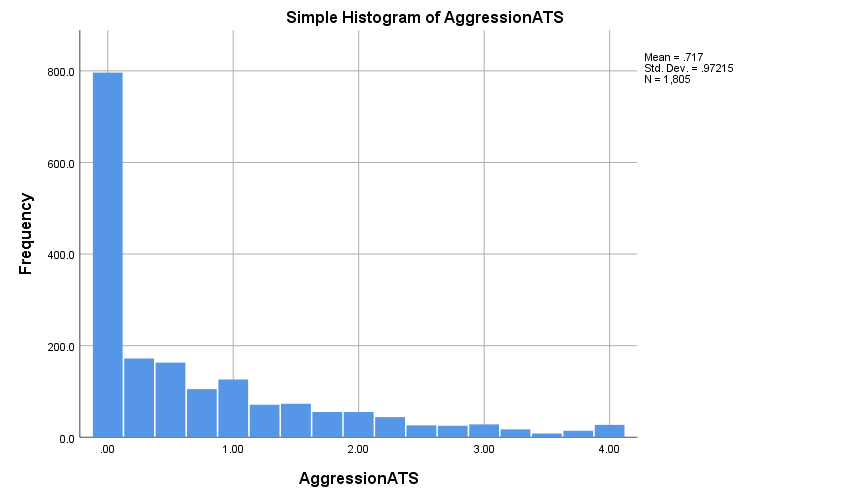


Supplementary Graph 5 Frequency distribution histogram for Averaged total score for aggression (AggressionATS)


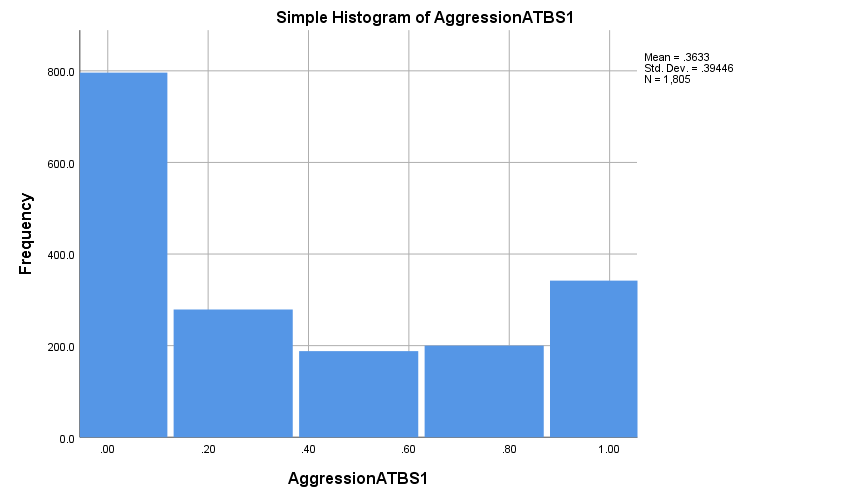


Supplementary Graph 6 Frequency distribution histogram for Averaged total binary score 1 for aggression (AggressionATBS1)


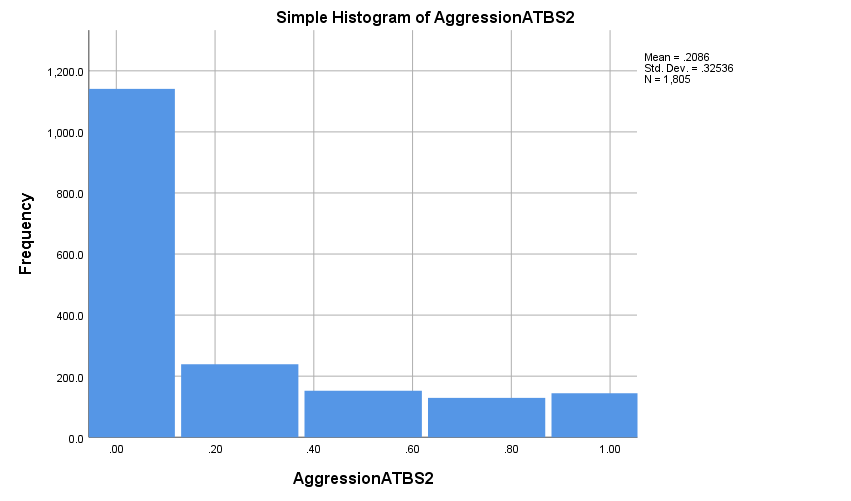


Supplementary Graph 7 Frequency distribution histogram for Averaged total binary score 2 for aggression (AggressionATBS2)


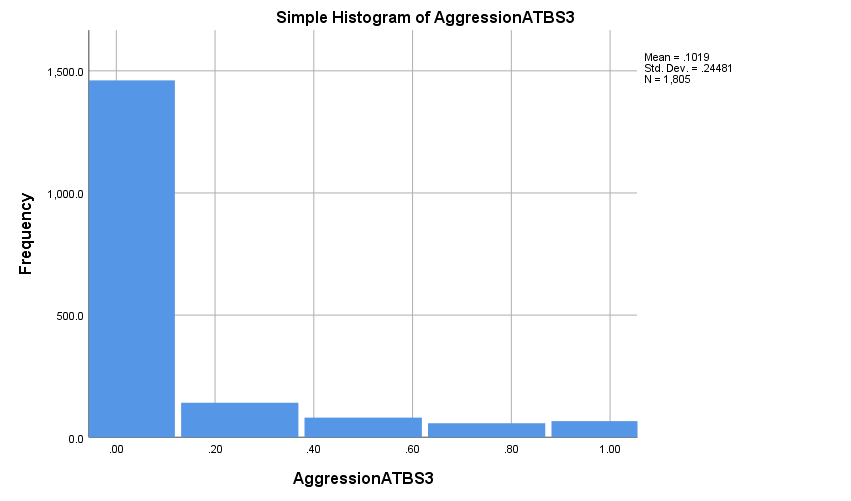


Supplementary Graph 8 Frequency distribution histogram for Averaged total binary score 3 for aggression (AggressionATBS3)


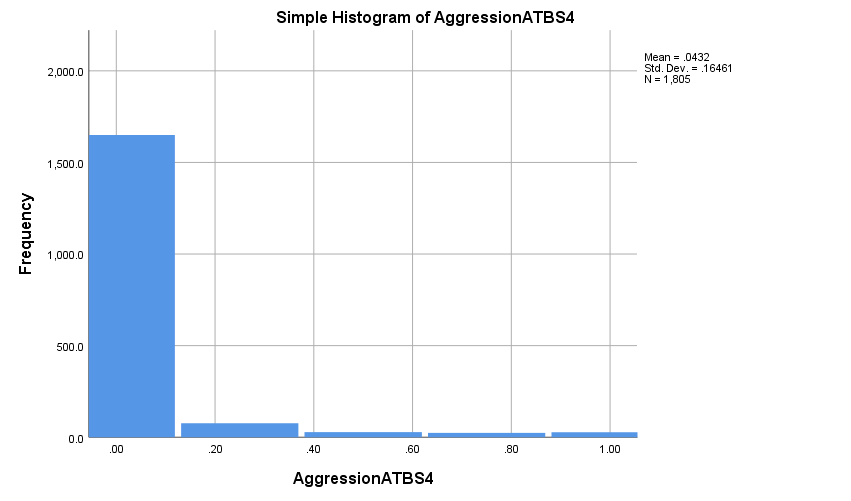


Supplementary Graph 9 Frequency distribution histogram for Averaged total binary score 4 for aggression (AggressionATBS4)


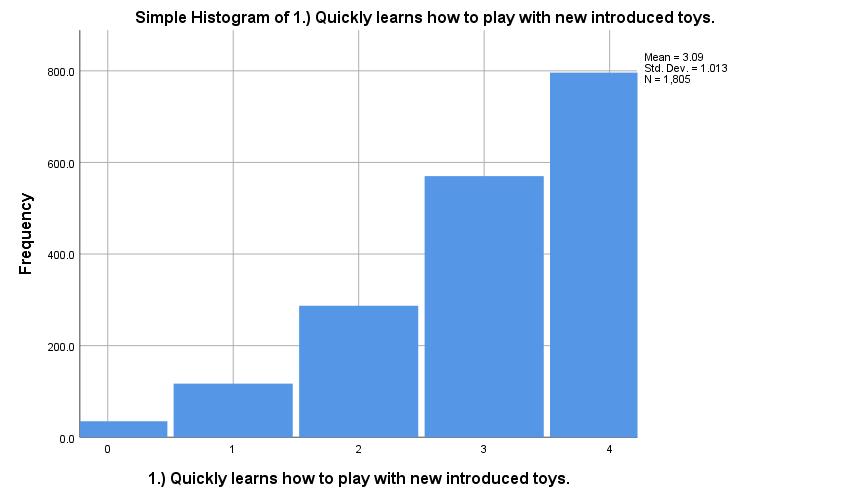


Supplementary Graph 10 Frequency distribution histogram for playfulness item 1.) Quickly learns how to play with new introduced toys, x axis- frequency of behaviour occurrence:0=never, 1=seldom, 2=sometimes, 3=usually, 4=always


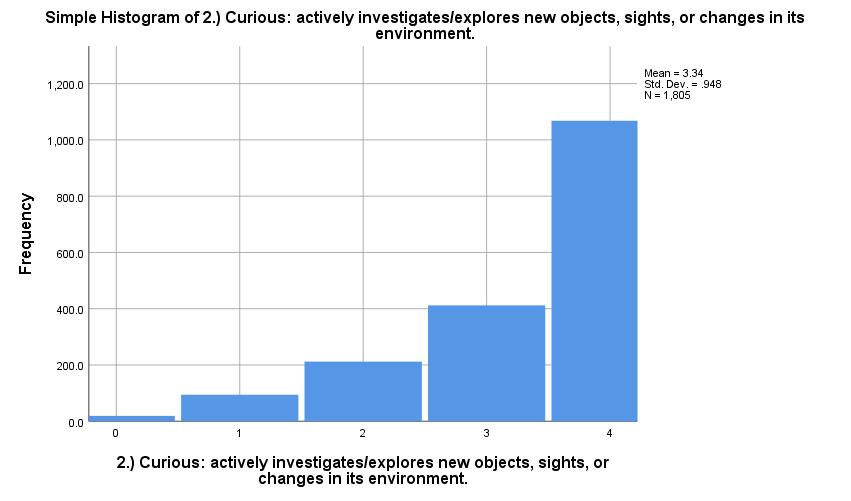


Supplementary Graph 11 Frequency distribution histogram for playfulness item 2.) Curious: actively investigates/explores new objects, sights, or changes in its environment, x axis- frequency of behaviour occurrence: 0=never, 1=seldom, 2=sometimes, 3=usually, 4=always


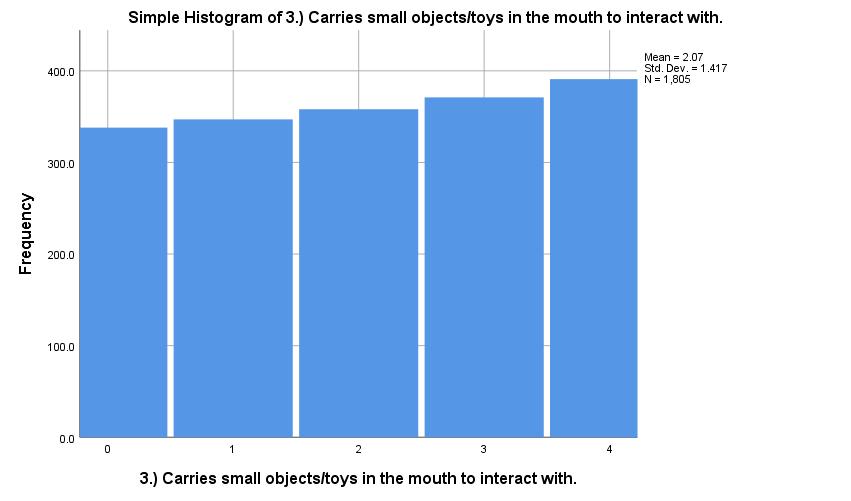


Supplementary Graph 12 Frequency distribution histogram for playfulness item 3.) Carries small objects/toys in the mouth to interact with, x axis- frequency of behaviour occurrence: 0=never, 1=seldom, 2=sometimes, 3=usually, 4=always


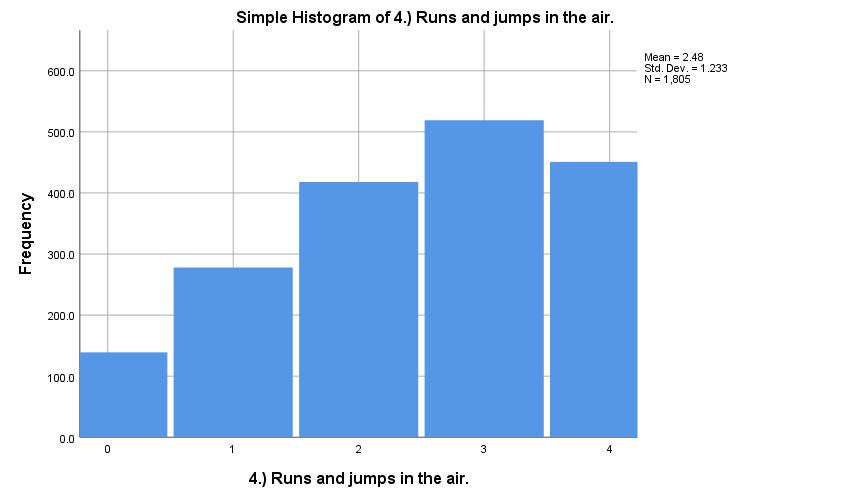


Supplementary Graph 13 Frequency distribution histogram for playfulness item 4.) Runs and jumps in the air, x axis- frequency of behaviour occurrence: 0=never, 1=seldom, 2=sometimes, 3=usually, 4=always


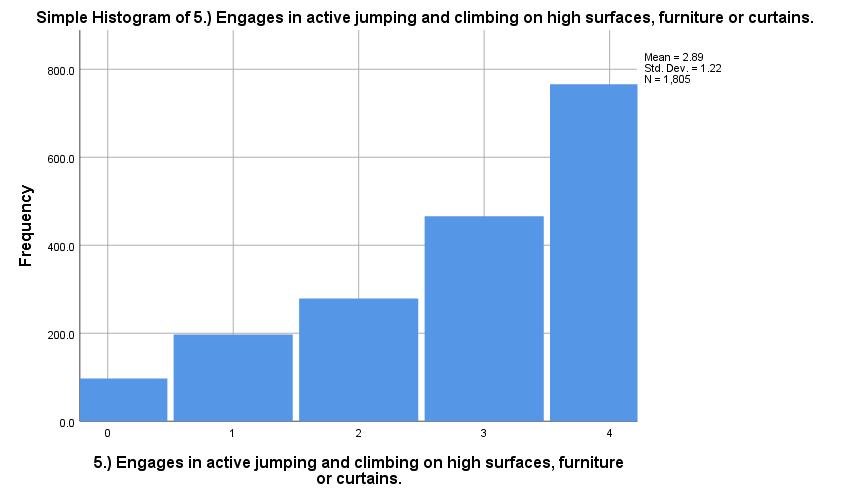


Supplementary Graph 14 Frequency distribution histogram for playfulness item 5.) Engages in active jumping and climbing on high surfaces, furniture or curtains, x axis- frequency of behaviour occurrence:0=never, 1=seldom, 2=sometimes, 3=usually, 4=always


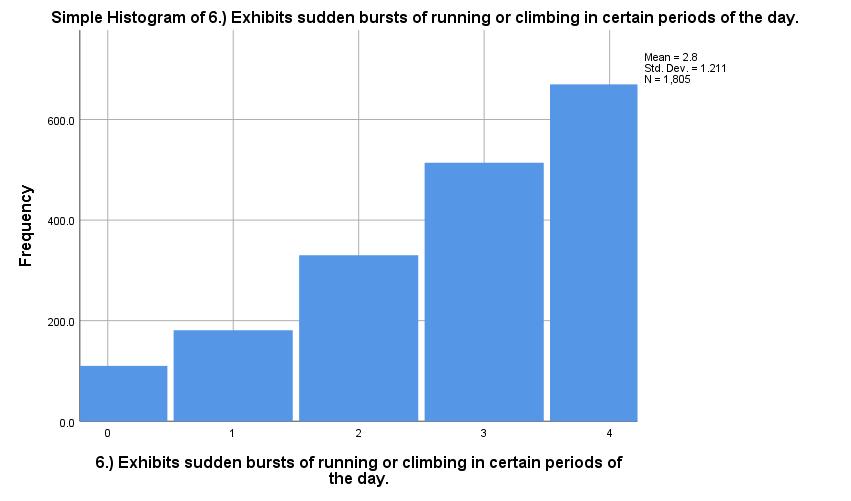


Supplementary Graph 15 Frequency distribution histogram for playfulness item 6.) Exhibits sudden bursts of running or climbing in certain periods of the day, x axis- frequency of behaviour occurrence:0=never, 1=seldom, 2=sometimes, 3=usually, 4=always


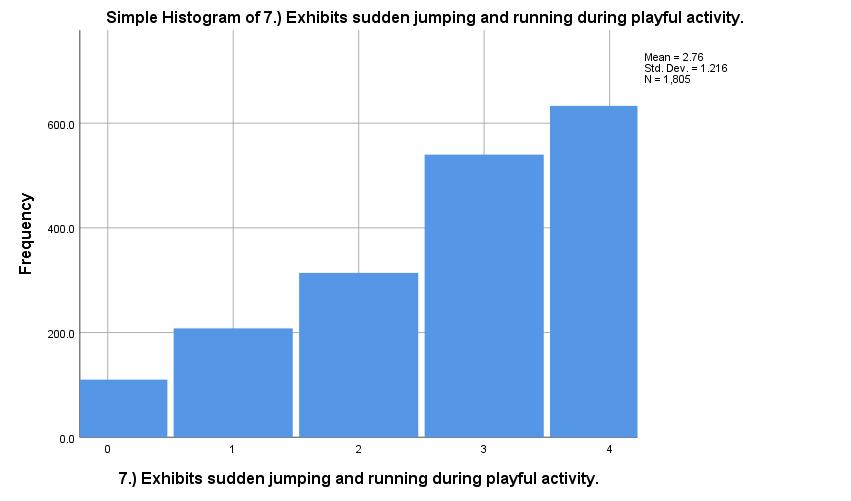


Supplementary Graph 16 Frequency distribution histogram for playfulness item 7.) Exhibits sudden jumping and running during playful activity, x axis- frequency of behaviour occurrence: 0=never, 1=seldom, 2=sometimes, 3=usually, 4=always


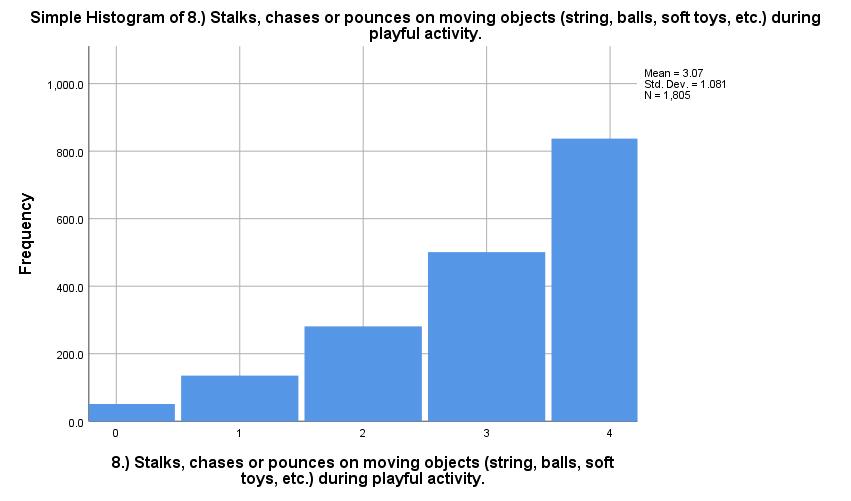


Supplementary Graph 17 Frequency distribution histogram for playfulness item 8.) Stalks, chases or pounces on moving objects (string, balls, soft toys, etc.) during playful activity, x axis- frequency of behaviour occurrence: 0=never, 1=seldom, 2=sometimes, 3=usually, 4=always


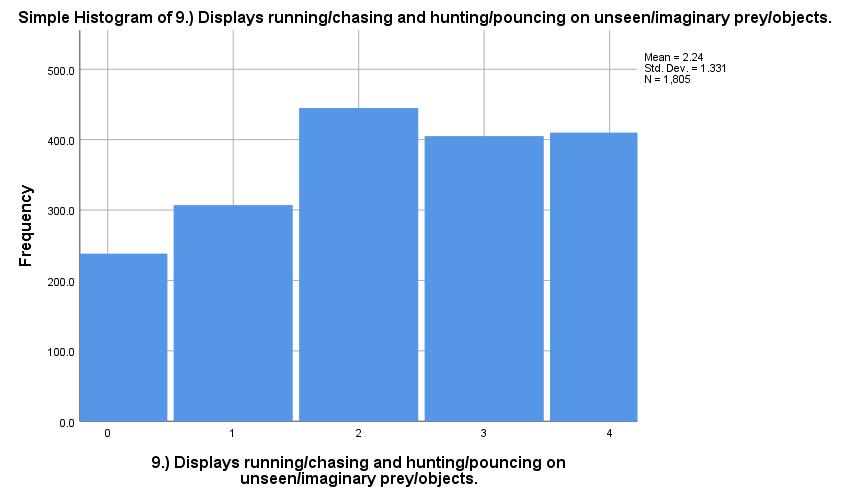


Supplementary Graph 18 Frequency distribution histogram for playfulness item 9.) Displays running/chasing and hunting/pouncing on unseen/imaginary prey/objects, x axis- frequency of behaviour occurrence: 0=never, 1=seldom, 2=sometimes, 3=usually, 4=always


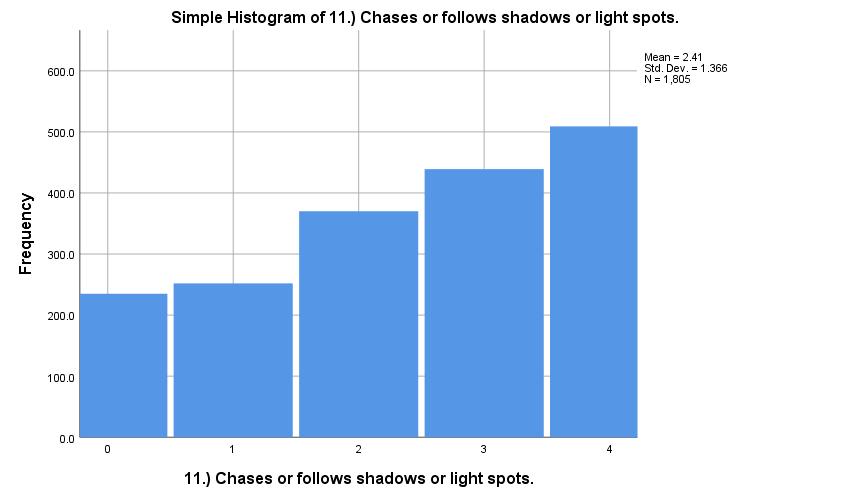


Supplementary Graph 19 Frequency distribution histogram for playfulness item 11.) Chases or follows shadows or light spots, x axis- frequency of behaviour occurrence: 0=never, 1=seldom, 2=sometimes, 3=usually, 4=always


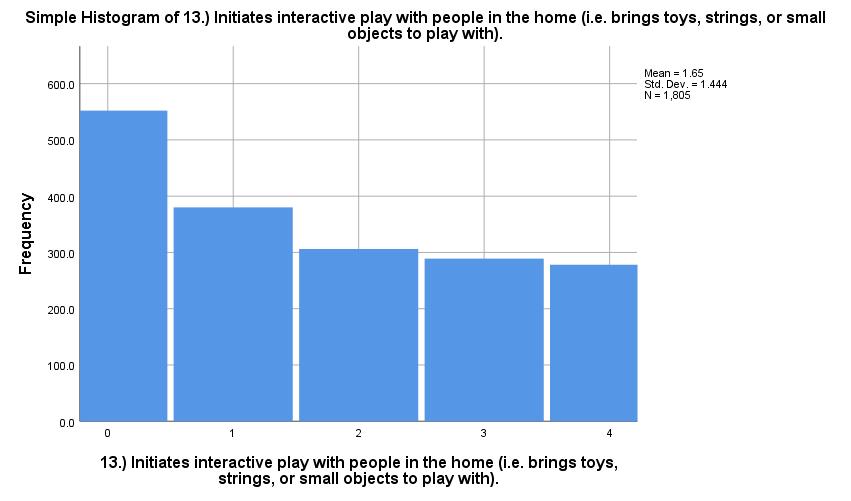


Supplementary Graph 20 Frequency distribution histogram for playfulness item 13.) Initiates interactive play with people in the home (i.e. brings toys, strings, or small objects to play with), x axis- frequency of behaviour occurrence: 0=never, 1=seldom, 2=sometimes, 3=usually, 4=always


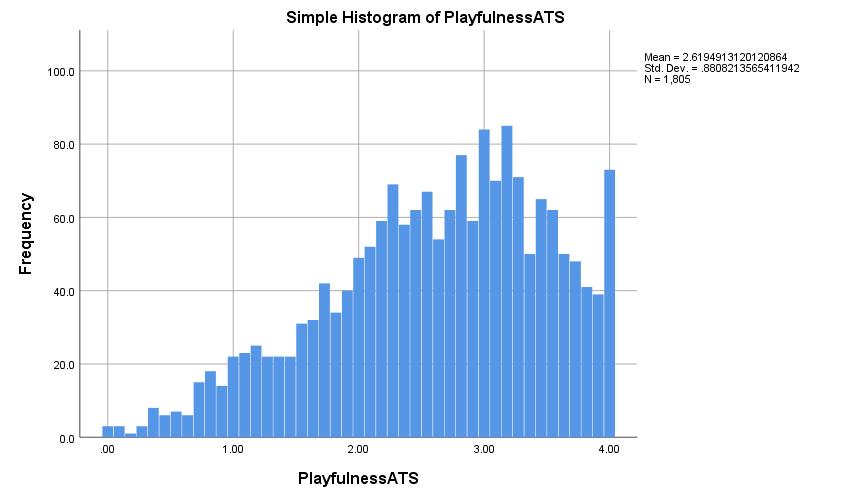


Supplementary Graph 21 Frequency distribution histogram for Averaged total score for playfulness (PlayfulnessATS)


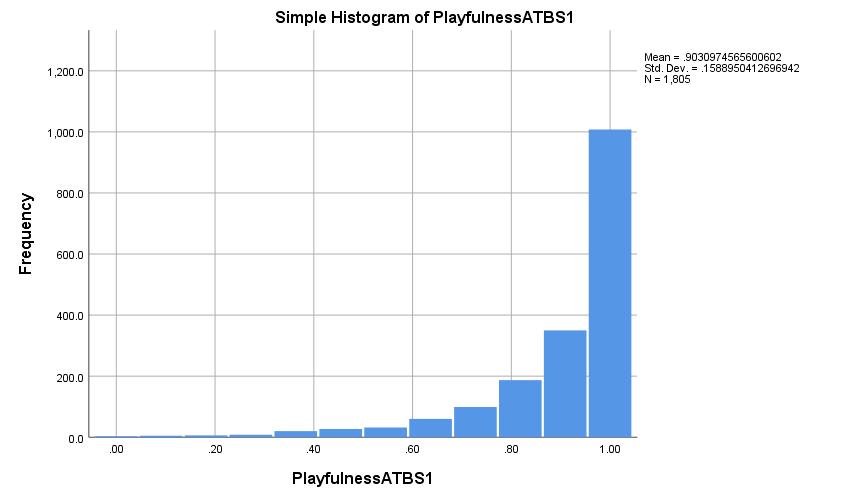


Supplementary Graph 22 Frequency distribution histogram for Averaged total binary score 1 for playfulness (PlayfulnessATBS1)


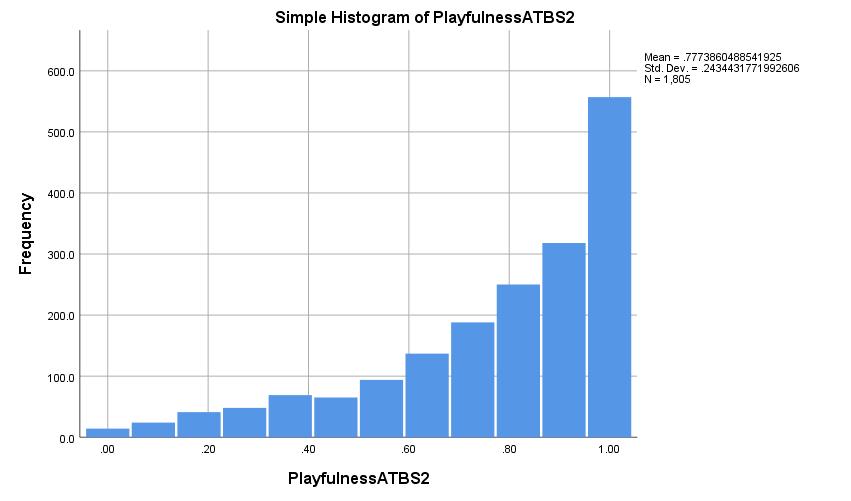


Supplementary Graph 23 Frequency distribution histogram for Averaged total binary score 2 for playfulness (PlayfulnessATBS2)


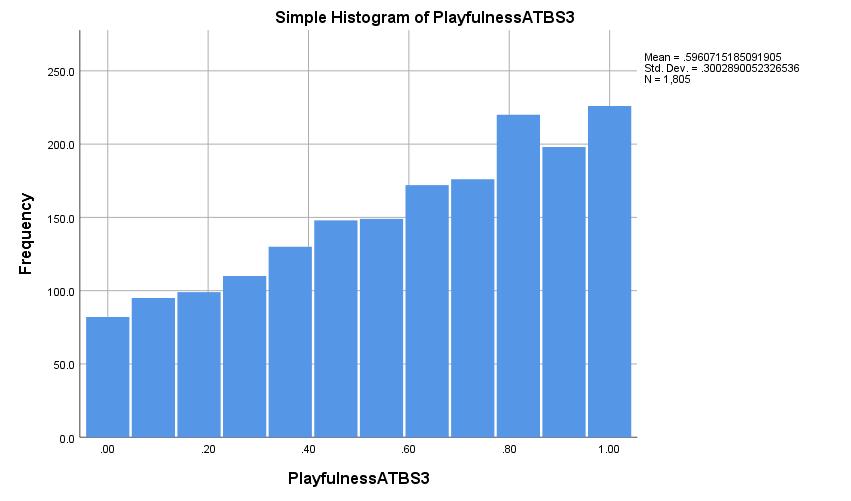


Supplementary Graph 24 Frequency distribution histogram for Averaged total binary score 3 for playfulness (PlayfulnessATBS3)


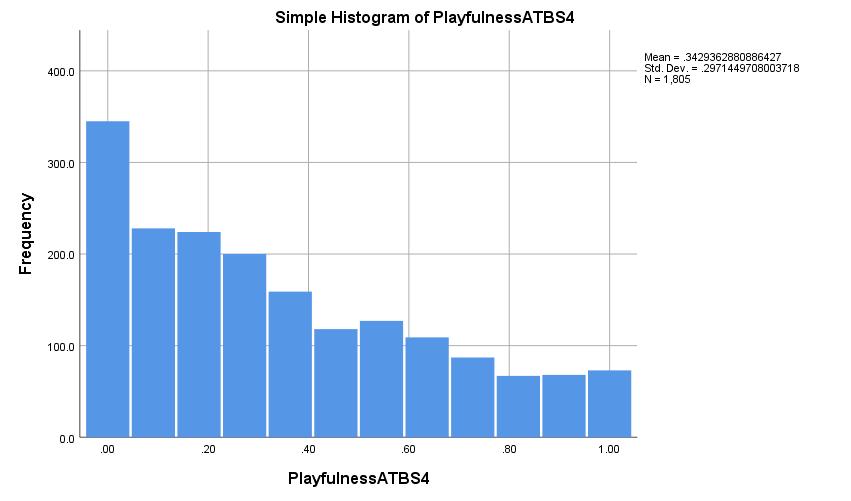


Supplementary Graph 25 Frequency distribution histogram for Averaged total binary score 4 for playfulness (PlayfulnessATBS4)

# Supplementary Tables

Supplementary Table 1 Risk factors for aggression using non-parametric tests of difference KW=Kruskal-Wallis test – H value, MW=Mann-Whitney test – U value, τ=Kendall´s tau correlation coefficient, Significant results are highlighted in the grey, * p≤0.05, ** p≤0.01, *** p≤0.001

| **Aggression - risk factors** | **Averaged total score** | **Averaged total binary score 1** | **Averaged total binary score 2** | **Averaged total binary score 3** | **Averaged total binary score 4** |
| --- | --- | --- | --- | --- | --- |
| Age (KW) | 130.338*** | 143.037*** | 89.017*** | 34.717*** | 16.649 |
| Breed type (KW) | 34.434*** | 33.704*** | 25.496*** | 20.686*** | 12.853** |
| Sex and neuter status (KW) | 65.704*** | 62.208*** | 56.994*** | 47.955*** | 39.689*** |
| Obtained from (KW) | 35.460*** | 36.187*** | 27.349*** | 20.878* | 11.725 |
| Age when obtained (KW) | 18.937*** | 20.693*** | 14.061** | 5.514 | 7.818 |
| Lifestyle (KW) | 22.739*** | 18.98*** | 28.528*** | 10.784* | 7.293 |
| Left alone (KW) | 3.456 | 1.819 | 6.138 | 3.576 | 0.706 |
| Dog (MW) | 364709.500 | 362869.500 | 368121.500 | 363196.500 | 368490.000 |
| Health (MW) | 230655.500** | 232174.000* | 238374.000 | 233507.500** | 246702.000 |
| Q Social play (τ) | -0.316*** | -0.308*** | -0.308*** | -0.277*** | -0.208*** |
| Q Fearfulness (τ) | 0.073*** | 0.068*** | 0.079*** | 0.068*** | 0.066*** |
| Q Scratching (τ) | 0.041* | 0.034 | 0.065*** | 0.063** | 0.065** |
| Q Spraying (τ) | 0.097*** | 0.105*** | 0.080*** | 0.055* | 0.022 |

Supplementary Table 2 Mean ranks of significant risk factors in Kruskal-Wallis (KW) and Mann-Whitney (MW) tests per each method of aggression score calculation, ATS= Averaged total score, ATBS1= Averaged total binary score 1, ATBS2= Averaged total binary score 2, ATBS3= Averaged total binary score 3, ATBS4= Averaged total binary score 4

| **Significant risk factors- Aggression** | **N** | **ATS** | **ATBS1** | **ATBS2** | **ATBS3** | **ATBS4** |
| --- | --- | --- | --- | --- | --- | --- |
| **Age (KW)** |  | | | | | |
| 6 months - 1 year | 113 | 609.51 | 604.74 | 697.96 | 808.70 | Not significant |
| 1 | 179 | 756.30 | 746.43 | 786.67 | 851.30 |  |
| 2 | 228 | 781.62 | 773.34 | 812.43 | 865.23 |  |
| 3 | 239 | 878.51 | 871.11 | 901.96 | 890.60 |  |
| 4 | 173 | 877.30 | 873.77 | 889.42 | 910.17 |  |
| 5 | 158 | 898.92 | 904.18 | 889.00 | 865.47 |  |
| 6 | 135 | 948.19 | 964.08 | 922.33 | 905.01 |  |
| 7 | 100 | 1065.16 | 1074.27 | 1037.38 | 965.88 |  |
| 8 | 82 | 1075.26 | 1076.95 | 1044.10 | 969.34 |  |
| 9 | 70 | 1106.51 | 1098.03 | 1099.95 | 1000.33 |  |
| 10 | 75 | 1055.71 | 1054.94 | 1041.56 | 946.73 |  |
| Over 11 | 253 | 1044.91 | 1057.54 | 972.78 | 958.66 |  |
| **Breed type - Mean ranks (KW)** |  | | | | | |
| Domestic short hair/mixed breed | 1046 | 951.00 | 950.88 | 936.83 | 932.37 | 917.95 |
| Domestic long hair/mixed breed | 275 | 895.21 | 892.56 | 906.38 | 892.18 | 900.88 |
| Purebreed | 379 | 776.10 | 779.04 | 801.49 | 837.72 | 863.58 |
| Mix of pure breeds (or if only one parent of your cat is pedigree) | 105 | 903.33 | 900.80 | 923.51 | 874.34 | 901.94 |
| **Sex and neuter status - mean ranks (KW)** |  | | | | | |
| Neutered Female | 854 | 1001.83 | 998.35 | 985.82 | 963.28 | 940.96 |
| Intact Female | 59 | 885.19 | 879.67 | 907.68 | 906.25 | 917.22 |
| Neutered Male | 844 | 812.41 | 817.26 | 823.80 | 845.97 | 863.73 |
| Intact Male | 48 | 759.40 | 742.91 | 816.46 | 829.21 | 900.66 |
| **Obtained from - mean ranks (KW)** |  | | | | | |
| Breeding cattery | 268 | 780.46 | 777.75 | 814.54 | 840.61 | Not significant |
| Cattery (boarding/foster) | 29 | 774.48 | 785.79 | 813.53 | 885.02 |  |
| Friend/relative/neighbour | 425 | 981.66 | 981.92 | 971.84 | 920.71 |  |
| Home (born at) | 98 | 822.08 | 827.16 | 847.04 | 840.40 |  |
| Pet store (purchase) | 42 | 1003.15 | 1002.06 | 993.36 | 1000.81 |  |
| Pet store (rescue) | 73 | 975.49 | 982.48 | 949.27 | 945.06 |  |
| Selling website | 100 | 919.89 | 892.34 | 934.10 | 943.09 |  |
| Shelter | 427 | 891.01 | 895.27 | 879.73 | 898.48 |  |
| Street (as a stray) | 303 | 921.59 | 922.97 | 912.93 | 932.28 |  |
| Veterinary hospital | 40 | 887.21 | 883.29 | 882.25 | 845.91 |  |
| **Age when obtained - mean ranks (KW)** |  | | | | | |
| Unweaned kitten (0 - 2 months) | 332 | 901.23 | 906.55 | 901.91 | Not significant | |
| Kitten (2 - 6 months) | 1042 | 880.79 | 877.14 | 886.96 |  |  |
| Junior (6 months - 2 years) | 282 | 902.04 | 908.91 | 895.43 |  |  |
| Mature (3 – 10 years) | 137 | 1077.57 | 1081.14 | 1038.76 |  |  |
| Senior (11+ years) | 12 | 909.96 | 877.50 | 953.54 |  |  |
| **Lifestyle (KW)** |  | | | | | |
| Indoors only | 821 | 855.64 | 861.86 | 854.55 | 880.25 | Not significant |
| Indoors with controlled or limited access to space outdoors (e.g. fenced garden or pen) | 494 | 895.82 | 892.45 | 896.77 | 900.16 |  |
| Indoors with free access outdoors | 481 | 989.77 | 982.57 | 989.45 | 943.89 |  |
| Outside only (no access indoors) | 7 | 1033.86 | 1038.36 | 1080.79 | 1011.00 |  |
| Lives in pen/stall/cage and has controlled access outdoors | 2 | 791.25 | 784.00 | 916.00 | 731.00 |  |
| **Is this cat currently suffering from any health problems? (MW)** |  | | | | | |
| No | 1459 | 888.09 | 889.13 | Not significant | 890.05 | Not significant |
| Yes | 346 | 965.87 | 961.48 |  | 957.62 |  |

Supplementary Table 3 Kendall´s tau correlation coefficients for Fe-BARQ single questions per each method of aggression score calculation, ATS= Averaged total score, ATBS1= Averaged total binary score 1, ATBS2= Averaged total binary score 2, ATBS3= Averaged total binary score 3, ATBS4= Averaged total binary score 4, * p≤0.05, ** p≤0.01, *** p≤0.001

| **Kendall´s tau -Aggression** |  | **Social play** | **Spraying** | **Fearfulness** | **Scratching** |
| --- | --- | --- | --- | --- | --- |
| **ATS** | Correlation Coefficient | -.0316*** | 0.097*** | 0.073*** | 0.041* |
|  | Sig. (2-tailed) | ≤0.001 | ≤0.001 | ≤0.001 | 0.029 |
|  | N | 1805 | 1805 | 1805 | 1805 |
| **ATBS1** | Correlation Coefficient | -0.308*** | 0.105*** | 0.068*** | 0.034 |
|  | Sig. (2-tailed) | ≤0.001 | ≤0.001 | ≤0.001 | 0.077 |
|  | N | 1805 | 1805 | 1805 | 1805 |
| **ATBS2** | Correlation Coefficient | -0.308*** | 0.080*** | 0.079*** | 0.065*** |
|  | Sig. (2-tailed) | ≤0.001 | ≤0.001 | ≤0.001 | 0.001 |
|  | N | 1805 | 1805 | 1805 | 1805 |
| **ATBS3** | Correlation Coefficient | -0.277*** | 0.055* | 0.068*** | 0.063** |
|  | Sig. (2-tailed) | ≤0.001 | 0.013 | 0.001 | 0.002 |
|  | N | 1805 | 1805 | 1805 | 1805 |
| **ATBS4** | Correlation Coefficient | -0.208*** | 0.022 | 0.066*** | 0.065** |
|  | Sig. (2-tailed) | ≤0.001 | 0.327 | 0.001 | 0.002 |
|  | N | 1805 | 1805 | 1805 | 1805 |

Supplementary Table 4 Risk factors for playfulness using non-parametric tests of difference KW=Kruskal-Wallis test – H value, MW=Mann-Whitney test – U value, τ=Kendall´s tau correlation coefficient, Significant results are highlighted in the grey, * p≤0.05, ** p≤0.01, *** p≤0.001

| **Playfulness - risk factors** | **Averaged total score** | **Averaged total binary score 1** | **Averaged total binary score 2** | **Averaged total binary score 3** | **Averaged total binary score 4** |
| --- | --- | --- | --- | --- | --- |
| Age (KW) | 399.761*** | 195.676*** | 324.708*** | 372.125*** | 317.545*** |
| Breed type (KW) | 56.960*** | 25.818*** | 44.817*** | 48.129*** | 51.798*** |
| Sex and neuter status (KW) | 35.063*** | 16.148*** | 22.143*** | 31.071*** | 34.931*** |
| Obtained from (KW) | 67.383*** | 43.053*** | 51.851*** | 65.186*** | 65.910*** |
| Age when obtained (KW) | 84.260*** | 48.036*** | 70.691*** | 76.127*** | 65.712*** |
| Lifestyle (KW) | 64.019*** | 49.111*** | 54.697*** | 47.929*** | 48.984*** |
| Left alone (KW) | 17.786*** | 11.751* | 16.423** | 17.837*** | 15.587** |
| Dog (MW) | 331247.000*** | 325325.000*** | 330435.000*** | 333990.000*** | 347066.500* |
| Health (MW) | 178475.000*** | 198084.500*** | 182016.000*** | 178953.000*** | 196456.500*** |
| Q Social play (τ) | 0.459*** | 0.377*** | 0.435*** | 0.435*** | 0.427*** |
| Q Fearfulness (τ) | -0.069*** | -0.073*** | -0.064*** | -0.063*** | -0.069*** |
| Q Scratching (τ) | 0.049** | 0.016 | 0.030 | 0.051** | 0.053** |
| Q Spraying (τ) | -0.019 | -0.027 | -0.021 | -0.020 | -0.008 |

Supplementary Table 5 Mean ranks of significant risk factors in Kruskal-Wallis (KW) and Mann-Whitney (MW) tests per each method of playfulness score calculation, ATS= Averaged total score, ATBS1= Averaged total binary score 1, ATBS= Averaged total binary score 2, ATBS3= Averaged total binary score 3, ATBS4= Averaged total binary score 4

| **Significant risk factors - Playfulness** | **N** | **ATS** | **ATBS1** | **ATBS2** | **ATBS3** | **ATBS4** |
| --- | --- | --- | --- | --- | --- | --- |
| **Age KW** |  | | | | | |
| 6 months - 1 year | 113 | 1368.38 | 1159.78 | 1263.84 | 1320.29 | 1364.00 |
| 1 | 179 | 1208.49 | 1087.09 | 1161.54 | 1204.84 | 1172.97 |
| 2 | 228 | 1098.86 | 1019.98 | 1083.28 | 1101.60 | 1059.45 |
| 3 | 239 | 1015.33 | 960.75 | 1002.87 | 1012.16 | 1001.95 |
| 4 | 173 | 925.33 | 916.12 | 940.44 | 934.27 | 913.26 |
| 5 | 158 | 819.70 | 889.49 | 864.82 | 829.13 | 819.59 |
| 6 | 135 | 870.93 | 923.06 | 885.81 | 863.97 | 862.65 |
| 7 | 100 | 778.96 | 928.85 | 838.30 | 763.72 | 750.32 |
| 8 | 82 | 676.53 | 693.40 | 717.61 | 704.18 | 705.11 |
| 9 | 70 | 701.93 | 788.77 | 719.16 | 680.89 | 731.81 |
| 10 | 75 | 668.04 | 720.13 | 670.56 | 694.75 | 701.81 |
| Over 11 | 253 | 497.97 | 630.39 | 514.95 | 509.23 | 569.74 |
| **Breed type KW** |  | | | | | |
| Domestic short hair/mixed breed | 1046 | 870.78 | 882.13 | 874.76 | 873.46 | 875.05 |
| Domestic long hair/mixed breed | 275 | 805.04 | 837.23 | 825.20 | 810.92 | 806.51 |
| Purebreed | 379 | 1076.94 | 1006.80 | 1055.10 | 1061.83 | 1066.49 |
| Mix of pure breeds (or if only one parent of your cat is pedigree) | 105 | 852.70 | 908.44 | 839.07 | 865.16 | 844.02 |
| **Sex and neuter status KW** |  | | | | | |
| Neutered Female | 854 | 867.89 | 868.21 | 876.99 | 869.42 | 871.66 |
| Intact Female | 59 | 1268.39 | 1094.86 | 1197.98 | 1247.77 | 1248.67 |
| Neutered Male | 844 | 906.76 | 923.43 | 906.77 | 907.90 | 900.62 |
| Intact Male | 48 | 1012.29 | 926.90 | 936.83 | 990.58 | 1077.53 |
| **Obtained from KW** |  | | | | | |
| Breeding cattery | 268 | 1104.82 | 1019.61 | 1075.27 | 1094.8 | 1100.54 |
| Cattery (boarding/foster) | 29 | 1105.52 | 1189.33 | 1058.22 | 1085.31 | 1091.98 |
| Friend/relative/neighbour | 425 | 840.10 | 859.59 | 852.23 | 836.78 | 851.78 |
| Home (born at) | 98 | 954.67 | 922.90 | 929.78 | 969.76 | 986.06 |
| Pet store (purchase) | 42 | 661.30 | 760.76 | 707.27 | 656.93 | 647.86 |
| Pet store (rescue) | 73 | 891.34 | 978.18 | 930.23 | 864.80 | 853.45 |
| Selling website | 100 | 948.53 | 907.20 | 936.29 | 967.26 | 925.95 |
| Shelter | 427 | 877.35 | 900.23 | 895.39 | 877.60 | 857.75 |
| Street (as a stray) | 303 | 844.31 | 835.38 | 826.59 | 860.38 | 863.25 |
| Veterinary hospital | 40 | 825.29 | 870.01 | 842.74 | 787.15 | 868.21 |
| **Age when obtained - mean ranks KW** |  | | | | | |
| Unweaned kitten (0 - 2 months) | 332 | 933.39 | 928.69 | 920.31 | 941.59 | 930.39 |
| Kitten (2 - 6 months) | 1042 | 962.83 | 937.11 | 955.60 | 956.76 | 960.37 |
| Junior (6 months - 2 years) | 282 | 821.57 | 866.77 | 849.96 | 825.90 | 801.16 |
| Mature (3 – 10 years) | 137 | 588.78 | 701.95 | 617.50 | 602.77 | 643.67 |
| Senior (11+ years) | 12 | 368.21 | 376.67 | 362.54 | 406.13 | 517.54 |
| **Lifestyle - mean ranks KW** |  | | | | | |
| Indoors only | 821 | 952.74 | 934.87 | 939.81 | 945.88 | 952.83 |
| Indoors with controlled or limited access to space outdoors (e.g. fenced garden or pen) | 494 | 971.65 | 968.93 | 978.77 | 960.67 | 952.57 |
| Indoors with free access outdoors | 481 | 751.73 | 784.20 | 767.72 | 775.25 | 767.76 |
| Outside only (no access indoors) | 7 | 459.14 | 561.71 | 458.29 | 447.43 | 681.21 |
| Lives in pen/stall/cage and has controlled access outdoors | 2 | 1463.00 | 1301.50 | 1166.25 | 1376.00 | 1505.00 |
| **Left alone per weak - mean ranks KW** |  | | | | | |
| 0 to 6 hours | 720 | 949.94 | 914.74 | 943.07 | 948.98 | 952.58 |
| 7 to 5 hours | 190 | 863.29 | 861.18 | 864.64 | 868.29 | 871.62 |
| 16 to 29 hours | 450 | 910.44 | 929.54 | 895.91 | 907.67 | 906.49 |
| 30 to 42 hours | 360 | 858.89 | 903.35 | 894.84 | 863.95 | 834.86 |
| 43 and more hours | 85 | 741.58 | 755.06 | 721.39 | 731.78 | 823.25 |
| **Does a dog live in your household? - mean ranks MW** |  | | | | | |
| No | 1180 | 934.78 | 939.8 | 935.47 | 932.46 | 921.38 |
| Yes | 625 | 843.00 | 833.52 | 841.70 | 847.38 | 868.31 |
| **Is this cat currently suffering from any health problems? MW** |  | | | | | |
| No | 1459 | 953.67 | 940.23 | 951.25 | 953.35 | 941.35 |
| Yes | 346 | 689.32 | 746.00 | 699.56 | 690.71 | 741.29 |

Supplementary Table 6 Kendall´s tau correlation coefficients for Fe-BARQ single questions per each method of playfulness score calculation, ATS= Averaged total score, ATBS1= Averaged total binary score 1, ATBS2= Averaged total binary score 2, ATBS3= Averaged total binary score, ATBS4= Averaged total binary score 4, * p≤0.05, ** p≤0.01, *** p≤0.001

| **Kendall´s tau -Playfulness** |  | **Social play** | **Spraying** | **Fearfulness** | **Scratching** |
| --- | --- | --- | --- | --- | --- |
| **ATS** | Correlation Coefficient | 0.459*** | -0.019 | -0.069*** | 0.049** |
|  | Sig. (2-tailed) | ≤0.001 | 0.310 | ≤0.001 | 0.005 |
|  | N | 1805 | 1805 | 1805 | 1805 |
| **ATBS1** | Correlation Coefficient | 0.377*** | -0.027 | -0.073*** | 0.016 |
|  | Sig. (2-tailed) | ≤0.001 | 0.204 | ≤0.001 | 0.414 |
|  | N | 1805 | 1805 | 1805 | 1805 |
| **ATBS2** | Correlation Coefficient | 0.420*** | -0.021 | -0.064*** | 0.030 |
|  | Sig. (2-tailed) | ≤0.001 | 0.290 | 0.001 | 0.110 |
|  | N | 1805 | 1805 | 1805 | 1805 |
| **ATBS3** | Correlation Coefficient | 0.435*** | -0.020 | -0.063*** | 0.051** |
|  | Sig. (2-tailed) | ≤0.001 | 0.299 | ≤0.001 | 0.004 |
|  | N | 1805 | 1805 | 1805 | 1805 |
| **ATBS4** | Correlation Coefficient | 0.427*** | -0.008 | -0.069*** | 0.053** |
|  | Sig. (2-tailed) | ≤0.001 | 0.682 | ≤0.001 | 0.003 |
|  | N | 1805 | 1805 | 1805 | 1805 |

Supplementary Table 7 Risk factors for aggression in subgroup of cats 3 years old and older (N=1285) using non-parametric tests of difference KW=Kruskal-Wallis test – H value, MW=Mann-Whitney test – U value, τ=Kendall´s tau correlation coefficient, Significant results are highlighted in the grey, * p≤0.05, ** p≤0.01, *** p≤0.001

| **Aggression - risk factors** | **Averaged total score** | **Averaged total binary score 1** | **Averaged total binary score 2** | **Averaged total binary score 3** | **Averaged total binary score 4** |
| --- | --- | --- | --- | --- | --- |
| Age (KW) | 37.359*** | 41.138*** | 25.986*** | 14.225 | 5.990 |
| Breed type (KW) | 23.136*** | 19.405*** | 21.895*** | 18.593*** | 10.345* |
| Sex and neuter status (KW) | 59.164*** | 53.099*** | 52.227*** | 46.189*** | 43.500*** |
| Obtained from (KW) | 24.021** | 22.529** | 21.782** | 18.584* | 8.374 |
| Age when obtained (KW) | 9.023 | 9.409 | 7.567 | 3.065 | 5.934 |
| Lifestyle (KW) | 13.714** | 9.761* | 19.209*** | 7.513 | 2.085 |
| Left alone (KW) | 2.025 | 1.091 | 3.419 | 1.841 | 1.486 |
| Dog (MW) | 180153.500 | 178401.500 | 183900.000 | 185895.000 | 184296.000 |
| Health (MW) | 143646.500 | 145491.500 | 144421.500 | 138922.000 | 144096.000 |
| Q Social play (τ) | ***-0.297 | ***-0.276 | ***-0.298 | ***-0.285 | ***-0.217 |
| Q Fearfulness (τ) | ***0.072 | **0.065 | ***0.087 | **0.064 | *0.056 |
| Q Scratching (τ) | *0.049 | 0.044 | **0.066 | *0.059 | *0.055 |
| Q Spraying (τ) | **0.073 | ***0.082 | *0.058 | 0.041 | 0.003 |

Supplementary Table 8 Mean ranks of significant risk factors for aggression in group of cats aged 3 years and older (N=1285) in Kruskal-Wallis (KW) and Mann-Whitney (MW) tests per each method of aggression score calculation, ATS= Averaged total score, ATBS1= Averaged total binary score, ATBS2= Averaged total binary score 2, ATBS3= Averaged total binary score 3, ATBS4= Averaged total binary score 4

| **Significant risk factors - Aggression** | **N** | **ATS** | **ATBS1** | **ATBS2** | **ATBS3** | **ATBS4** |
| --- | --- | --- | --- | --- | --- | --- |
| **Age (KW)** |  | | | | | |
| 3 | 239 | 577.27 | 569.38 | 605.03 | Not significant | Not significant |
| 4 | 173 | 576.81 | 572.27 | 597.73 |  |  |
| 5 | 158 | 589.76 | 591.18 | 597.68 |  |  |
| 6 | 135 | 627.79 | 637.73 | 621.91 |  |  |
| 7 | 100 | 713.70 | 717.97 | 703.62 |  |  |
| 8 | 82 | 716.76 | 714.76 | 707.47 |  |  |
| 9 | 70 | 742.35 | 732.73 | 745.41 |  |  |
| 10 | 75 | 702.08 | 698.50 | 703.56 |  |  |
| Over 11 | 253 | 694.87 | 701.92 | 658.24 |  |  |
| **Breed type - Mean ranks (KW)** |  |  |  |  |  |  |
| Domestic short hair/mixed breed | 776 | 675.62 | 672.54 | 669.38 | 667.36 | 656.38 |
| Domestic long hair/mixed breed | 194 | 629.37 | 628.29 | 636.69 | 627.07 | 627.39 |
| Purebreed | 240 | 548.55 | 557.54 | 555.81 | 588.93 | 615.67 |
| Mix of pure breeds (or if only one parent of your cat is pedigree) | 75 | 643.03 | 648.93 | 665.38 | 605.17 | 632.44 |
| **Sex and neuter status - mean ranks (KW)** |  |  |  |  |  |  |
| Neutered Female | 634 | 719.51 | 715.05 | 708.52 | 693.55 | 677.61 |
| Intact Female | 17 | 655.85 | 680.65 | 627.82 | 623.59 | 616.76 |
| Neutered Male | 622 | 563.56 | 568.46 | 574.70 | 591.59 | 607.62 |
| Intact Male | 12 | 700.46 | 646.67 | 743.13 | 664.25 | 685.67 |
| **Obtained from - mean ranks (KW)** |  |  |  |  |  |  |
| Breeding cattery | 161 | 559.02 | 564.67 | 574.09 | 593.11 | Not significant |
| Cattery (boarding/foster) | 20 | 557.60 | 563.10 | 593.90 | 663.45 |  |
| Friend/relative/neighbour | 321 | 690.55 | 689.69 | 692.55 | 648.30 |  |
| Home (born at) | 64 | 566.13 | 568.48 | 588.75 | 573.47 |  |
| Pet store (purchase) | 38 | 715.34 | 710.99 | 704.08 | 718.08 |  |
| Pet store (rescue) | 54 | 739.25 | 731.69 | 726.37 | 711.07 |  |
| Selling website | 63 | 623.15 | 605.83 | 625.32 | 647.56 |  |
| Shelter | 320 | 638.41 | 638.78 | 630.12 | 652.27 |  |
| Street (as a stray | 214 | 642.41 | 646.51 | 633.80 | 652.83 |  |
| Veterinary hospital | 30 | 635.75 | 628.20 | 643.75 | 592.53 |  |
| **Lifestyle (KW)** |  |  |  |  |  |  |
| Indoors only | 544 | 612.07 | 617.40 | 609.92 | Not significant | Not significant |
| Indoors with controlled or limited access to space outdoors (e.g. fenced garden or pen) | 348 | 630.12 | 631.56 | 627.15 |  |  |
| Indoors with free access outdoors | 386 | 698.33 | 689.35 | 703.00 |  |  |
| Outside only (no access indoors) | 6 | 614.33 | 626.67 | 669.58 |  |  |
| Lives in pen/stall/cage and has controlled access outdoors | 1 | 765.50 | 751.50 | 836.00 |  |  |

Supplementary Table 9 Kendall´s tau correlation coefficients for Fe-BARQ single questions per each method of aggression score calculation in group of cats aged 3 years and older (N=1285), ATS= Averaged total score, ATBS1= Averaged total binary score 1, ATBS2= Averaged total binary score 2, ATBS3= Averaged total binary score 3, ATBS4= Averaged total binary score 4, * p≤0.05, ** p≤0.01, *** p≤0.001

| **Kendall´s tau - Aggression** | | **Social play** | **Spraying** | **Fearfulness** | **Scratching** |
| --- | --- | --- | --- | --- | --- |
| **ATS** | Correlation Coefficient | -0.297*** | 0.073** | 0.072*** | 0.049* |
|  | Sig. (2-tailed) | ≤0.001 | 0.002 | 0.001 | 0.025 |
|  | N | 1285 | 1285 | 1285 | 1285 |
| **ATBS1** | Correlation Coefficient | -0.276*** | 0.082*** | 0.065** | 0.044 |
|  | Sig. (2-tailed) | ≤0.001 | 0.001 | 0.005 | 0.053 |
|  | N | 1285 | 1285 | 1285 | 1285 |
| **ATBS2** | Correlation Coefficient | -0.298*** | 0.058* | 0.087*** | 0.066** |
|  | Sig. (2-tailed) | ≤0.001 | 0.021 | ≤0.001 | 0.004 |
|  | N | 1285 | 1285 | 1285 | 1285 |
| **ATBS3** | Correlation Coefficient | -0.285*** | 0.041 | 0.064** | 0.059* |
|  | Sig. (2-tailed) | ≤0.001 | 0.122 | 0.008 | 0.014 |
|  | N | 1285 | 1285 | 1285 | 1285 |
| **ATBS4** | Correlation Coefficient | -0.217*** | 0.003 | 0.056* | 0.055* |
|  | Sig. (2-tailed) | ≤0.001 | 0.896 | 0.024 | 0.026 |
|  | N | 1285 | 1285 | 1285 | 1285 |

Supplementary Table 10 F value and partial eta squared for minimal adequate models for different ways of aggression score calculation methods in group of cats aged 3 years old and older (N=1285), * p≤0.05, ** p≤0.01, *** p≤0.001. Bold items are significant across all models.

| **Aggression- Minimal Adequate Model** | **Averaged total score** | | **Averaged total binary score 1** | | **Averaged total binary score 2** | | **Averaged total binary score 3** | | **Averaged total binary score 4** | |
| --- | --- | --- | --- | --- | --- | --- | --- | --- | --- | --- |
|  | **F** | **Partial Eta Squared** | **F** | **Partial Eta Squared** | **F** | **Partial Eta Squared** | **F** | **Partial Eta Squared** | **F** | **Partial Eta Squared** |
| Age | x | x | 5.624* | 0.004 | x | x | x | x | x | x |
| Breed type | 3.365* | 0.008 | 3.261* | 0.008 | 2.919* | 0.007 | 3.033* | 0.007 | x | x |
| **Sex and neuter status** | 14.817*** | 0.034 | 12.705*** | 0.029 | 11.123*** | 0.026 | 8.631 | 0.020 | 6.834*** | 0.016 |
| **Q Social play** | 206.150*** | 0.139 | 95.033*** | 0.069 | 171.094*** | 0.118 | 165.647*** | 0.115 | 97.670*** | 0.071 |
| Q Fearfulness | 7.830** | 0.006 | 7.784** | 0.006 | 8.401** | 0.007 | x | x | x | x |
| Q Scratching | 11.452*** | 0.009 | x | x | 11.294*** | 0.009 | 13.076*** | 0.010 | 5.701* | 0.004 |
| Q Spraying | 8.575** | 0.007 | 11.753*** | 0.009 | 5.845* | 0.005 | x | x | x | x |
| *R^2^* | 0.217 | | 0.159 | | 0.187 | | 0.165 | | 0.101 | |
| *Adj. R^2^* | 0.211 | | 0.152 | | 0.180 | | 0.160 | | 0.097 | |

Supplementary Table 11 Relationship between aggression (in subgroup of cats 3 years old and older, N=1285) and sex including neuter status (Kruskal-Wallis test, KW),and between aggression and social play (Kendall´s tau correlation, τ), all H statistics and Kendall´s tau correlation coefficients are significant at p≤0.001 level, difference in in values compared with original sample (N=1805) is highlighted by bold italics

| **Aggression by Sex and neuter status (KW)** | | **Averaged total score** | **Averaged total binary score 1** | **Averaged total binary score 2** | **Averaged total binary score 3** | **Averaged total binary score 4** |
| --- | --- | --- | --- | --- | --- | --- |
| Kruskal-Wallis H | | 59.164 | 53.099 | 52.227 | 46.189 | 43.500 |
| Mean ranks | Neutered females | 719.51 | 715.05 | 708.52 | 693.55 | 677.61 |
|  | Intact female | 655.85 | 680.65 | 627.82 | 623.59 | 616.76 |
|  | Neutered male | ***563.56*** | ***568.46*** | ***574.70*** | ***591.59*** | ***607.62*** |
|  | Intact male | ***700.46*** | ***646.67*** | ***743.13*** | ***664.25*** | ***685.67*** |
| **Aggression by Social play and Fearfulness (τ)** | |  |  |  |  |  |
| Social play | | -0.297 | -0.276 | -0.298 | -0.285 | -0.217 |

Supplementary Table 12 Risk factors for playfulness in subgroup of cats 3 years old and older (N=1285) using non-parametric tests of difference KW=Kruskal-Wallis test – H value, MW=Mann-Whitney test – U value, τ=Kendall´s tau correlation coefficient, Significant results are highlighted in the grey, * p≤0.05, ** p≤0.01, *** p≤0.001

| **Playfulness - risk factors** | **Averaged total score** | **Averaged total binary score 1** | **Averaged total binary score 2** | **Averaged total binary score 3** | **Averaged total binary score 4** |
| --- | --- | --- | --- | --- | --- |
| Age (KW) | 164.143*** | 89.671*** | 149.950*** | 156.950*** | 113.652*** |
| Breed type (KW) | 34.656*** | 17.593*** | 29.942*** | 26.682*** | 33.381*** |
| Sex and neuter status (KW) | 2.799 | 6.48 | 2.833 | 1.996 | 2.153 |
| Obtained from (KW) | 46.111*** | 33.015*** | 38.631*** | 40.237*** | 45.295*** |
| Age when obtained (KW) | 34.024*** | 24.583*** | 31.676*** | 29.847*** | 19.356*** |
| Lifestyle (KW) | 32.744*** | 31.758*** | 34.094*** | 22.237*** | 19.865*** |
| Left alone (KW) | 10.223 | 11.653* | 13.235** | 11.140* | 4.682 |
| Dog (MW) | 165278.500*** | 161930.500*** | 164338.500*** | 167325.500** | 176842.000 |
| Health (MW) | 111091.500*** | 119028.000*** | 113013.000*** | 110456.000*** | 123381.000** |
| Q Social play (τ) | ***0.434 | ***0.372 | ***0.410 | ***0.409 | ***0.380 |
| Q Fearfulness (τ) | **-0.055 | *-0.056 | *-0.050 | **-0.059 | **-0.056 |
| Q Scratching (τ) | 0.028 | 0.018 | 0.012 | 0.032 | 0.032 |
| Q Spraying (τ) | 0.009 | -0.008 | 0.006 | 0.006 | 0.026 |

Supplementary Table 13 Mean ranks of significant risk factors for playfulness in group of cats aged 3 years and older (N=1285) in Kruskal-Wallis (KW) and Mann-Whitney (MW) tests per each method of aggression score calculation, ATS= Averaged total score, ATBS1= Averaged total binary score 1, ATBS2= Averaged total binary score 2, ATBS3= Averaged total binary score 3, ATBS4= Averaged total binary score 4

| **Significant risk factors - Playfulness** | **N** | **ATS** | **ATBS1** | **ATBS2** | **ATBS3** | **ATBS4** |
| --- | --- | --- | --- | --- | --- | --- |
| **Age KW** |  | | | | | |
| 3 | 239 | 808.75 | 734.50 | 786.65 | 803.13 | 790.77 |
| 4 | 173 | 749.53 | 702.32 | 743.44 | 753.70 | 730.58 |
| 5 | 158 | 674.40 | 682.56 | 686.83 | 678.84 | 667.68 |
| 6 | 135 | 709.27 | 707.11 | 704.43 | 700.69 | 690.24 |
| 7 | 100 | 643.24 | 708.63 | 669.44 | 625.67 | 608.56 |
| 8 | 82 | 571.78 | 545.73 | 585.98 | 591.65 | 579.29 |
| 9 | 70 | 580.04 | 611.94 | 586.80 | 559.49 | 591.29 |
| 10 | 75 | 555.89 | 565.17 | 546.59 | 572.87 | 571.45 |
| Over 11 | 253 | 424.84 | 494.34 | 430.62 | 430.26 | 472.68 |
| **Breed type KW** |  |  |  |  |  |  |
| Domestic short hair/mixed breed | 776 | 627.68 | 630.24 | 630.71 | 630.79 | 631.26 |
| Domestic long hair/mixed breed | 194 | 558.54 | 594.22 | 565.81 | 566.30 | 552.74 |
| Purebreed | 240 | 758.43 | 722.49 | 749.76 | 742.75 | 751.04 |
| Mix of pure breeds (or if only one parent of your cat is pedigree) | 75 | 650.61 | 646.79 | 628.19 | 648.53 | 652.21 |
| **Obtained from KW** |  |  |  |  |  |  |
| Breeding cattery | 161 | 786.82 | 739.06 | 773.01 | 769.74 | 786.73 |
| Cattery (boarding/foster) | 20 | 843.65 | 857.95 | 770.25 | 822.43 | 840.78 |
| Friend/relative/neighbour | 321 | 603.74 | 609.93 | 607.83 | 601.59 | 614.47 |
| Home (born at) | 64 | 671.84 | 612.84 | 668.73 | 699.36 | 699.19 |
| Pet store (purchase) | 38 | 500.61 | 551.34 | 528.39 | 507.26 | 475.62 |
| Pet store (rescue) | 54 | 668.02 | 713.97 | 694.45 | 645.98 | 637.76 |
| Selling website | 63 | 694.45 | 673.01 | 687.59 | 712.24 | 657.91 |
| Shelter | 320 | 633.55 | 646.84 | 643.19 | 631.29 | 617.23 |
| Street (as a stray) | 214 | 594.28 | 601.74 | 582.30 | 608.50 | 611.63 |
| Veterinary hospital= | 30 | 571.60 | 581.1 | 571.72 | 558.23 | 614.10 |
| **Age when obtained - mean ranks KW** |  |  |  |  |  |  |
| Unweaned kitten (0 - 2 months) | 218 | 657.66 | 643.15 | 648.78 | 666.97 | 660.15 |
| Kitten (2 - 6 months) | 688 | 668.89 | 663.98 | 667.56 | 663.5 | 665.93 |
| Junior (6 months - 2 years) | 230 | 654.24 | 654.10 | 660.30 | 655.44 | 633.91 |
| Mature (3 – 10 years) | 137 | 500.15 | 548.60 | 511.18 | 507.86 | 534.58 |
| Senior (11+ years) | 12 | 307.79 | 302.67 | 303.13 | 336.38 | 428.63 |
| **Lifestyle - mean ranks KW** |  |  |  |  |  |  |
| Indoors only | 544 | 675.58 | 667.23 | 666.75 | 671.11 | 672.08 |
| Indoors with controlled or limited access to space outdoors (e.g. fenced garden or pen) | 348 | 686.80 | 693.11 | 699.5 | 674.11 | 670.81 |
| Indoors with free access outdoors | 386 | 559.97 | 565.58 | 561.01 | 578.22 | 576.90 |
| Outside only (no access indoors) | 6 | 405.17 | 466.00 | 406.67 | 377.17 | 566.83 |
| Lives in pen/stall/cage and has controlled access outdoors | 1 | 1152.00 | 968.00 | 1128.00 | 1122.00 | 1117.00 |
| **Left alone per weak - mean ranks KW** |  |  |  |  |  |  |
| 0 to 6 hours | 494 | Not significant | 650.26 | 664.39 | 668.01 | Not significant |
| 7 to 5 hours | 131 |  | 590.15 | 600.50 | 613.61 |  |
| 16 to 29 hours | 319 |  | 657.30 | 636.54 | 638.98 |  |
| 30 to 42 hours | 273 |  | 665.51 | 664.92 | 648.06 |  |
| 43 and more hours | 68 |  | 534.60 | 511.73 | 516.48 |  |
| **Does a dog live in your household? - mean ranks MW** |  |  |  |  |  |  |
| No | 838 | 669.27 | 673.27 | 670.39 | 666.83 | Not significant |
| Yes | 447 | 593.75 | 586.26 | 591.65 | 598.33 |  |
| **Is this cat currently suffering from any health problems? MW** |  | | | | | |
| No | 989 | 678.67 | 670.65 | 676.73 | 679.32 | 666.25 |
| Yes | 296 | 523.81 | 550.62 | 530.30 | 521.66 | 565.33 |

Supplementary Table 14 Kendall´s tau correlation coefficients for Fe-BARQ single questions per each method of playfulness score calculation in group of cats aged 3 years and older (N=1285), ATS= Averaged total score, ATBS1= Averaged total binary score 1, ATBS2= Averaged total binary score 2, ATBS3= Averaged total binary score 3, ATBS4= Averaged total binary score 4, * p≤0.05, ** p≤0.01, *** p≤0.001

| **Kendall´s tau - Playfulness** | | **Social play** | **Spraying** | **Fearfulness** | **Scratching** |
| --- | --- | --- | --- | --- | --- |
| **ATS** | Correlation Coefficient | 0.434*** | 0.009 | -0.055** | 0.028 |
|  | Sig. (2-tailed) | ≤0.001 | 0.692 | 0.009 | 0.183 |
|  | N | 1285 | 1285 | 1285 | 1285 |
| **ATBS1** | Correlation Coefficient | 0.372*** | -0.008 | -0.056* | 0.018 |
|  | Sig. (2-tailed) | ≤0.001 | 0.739 | 0.015 | 0.431 |
|  | N | 1285 | 1285 | 1285 | 1285 |
| **ATBS2** | Correlation Coefficient | 0.410*** | 0.006 | -0.050* | 0.012 |
|  | Sig. (2-tailed) | ≤0.001 | 0.813 | 0.022 | 0.596 |
|  | N | 1285 | 1285 | 1285 | 1285 |
| **ATBS3** | Correlation Coefficient | 0.409*** | 0.006 | -0.059** | 0.032 |
|  | Sig. (2-tailed) | ≤0.001 | 0.795 | 0.006 | 0.133 |
|  | N | 1285 | 1285 | 1285 | 1285 |
| **ATBS4** | Correlation Coefficient | 0.380*** | 0.026 | -0.056** | 0.032 |
|  | Sig. (2-tailed) | ≤0.001 | 0.264 | 0.010 | 0.146 |
|  | N | 1285 | 1285 | 1285 | 1285 |

Supplementary Table 15 F value and partial eta squared for minimal adequate models for different ways of playfulness score calculation methods in subgroup of cats 3 years old and older (N=1285), * p≤0.05, ** p≤0.01, *** p≤0.001. Bold items are significant across all models.

| **Playfulness- Minimal Adequate Model** | **Averaged total score** | | **Averaged total binary score 1** | | **Averaged total binary score 2** | | **Averaged total binary score 3** | | **Averaged total binary score 4** | |
| --- | --- | --- | --- | --- | --- | --- | --- | --- | --- | --- |
|  | **F** | **Partial Eta Squared** | **F** | **Partial Eta Squared** | **F** | **Partial Eta Squared** | **F** | **Partial Eta Squared** | **F** | **Partial Eta Squared** |
| **Age** | 50.175*** | 0.038 | 12.431*** | 0.010 | 46.931*** | 0.035 | 50.297*** | 0.038 | 25.507*** | 0.020 |
| Breed type | 8.104*** | 0.019 | x | x | 6.469*** | 0.015 | 5.813*** | 0.013 | 10.014*** | 0.023 |
| Obtained from | x | x | 2.637** | 0.018 | x | x | x | x | x | x |
| **Age when obtained** | 7.306** | 0.006 | 5.622* | 0.004 | 4.922* | 0.004 | 6.342* | 0.005 | 4.438* | 0.003 |
| Dog | 9.26** | 0.007 | 9.186** | 0.007 | 9.719** | 0.008 | 8.138** | 0.006 | x | x |
| Health | 8.639** | 0.007 | 10.208*** | 0.008 | 7.329** | 0.006 | 9.278** | 0.007 | x | x |
| **Q Social play** | 399.213*** | 0.238 | 233.481*** | 0.155 | 323.074*** | 0.202 | 303.117*** | 0.192 | 237.975*** | 0.157 |
| Q Fearfulness | 5.006* | 0.004 | x | x | x | x | 5.964* | 0.005 | x | x |
| *R^2^* | 0.373 | | 0.253 | | 0.327 | | 0.326 | | 0.246 | |
| *Adj.R^2^* | 0.368 | | 0.244 | | 0.323 | | 0.321 | | 0.242 | |

Supplementary Table 16 Relationship between playfulness (in subgroup of cats 3 years old and older, N=1285) and age, and age when obtained (Kruskal-Wallis test, KW) and between playfulness and social play Kendall´s tau correlation, τ), all H statistics and Kendall´s tau correlation coefficients are significant at p≤0.001 level.

| **Playfulness by Age (KW)** | | **Averaged total score** | **Averaged total binary score 1** | **Averaged total binary score 2** | **Averaged total binary score 3** | **Averaged total binary score 4** |
| --- | --- | --- | --- | --- | --- | --- |
| Kruskal-Wallis H | | 164.143 | 89.671 | 149.95 | 156.95 | 113.652 |
| Mean ranks | 3 | 808.75 | 734.5 | 786.65 | 803.13 | 790.77 |
|  | 4 | 749.53 | 702.32 | 743.44 | 753.7 | 730.58 |
|  | 5 | 674.4 | 682.56 | 686.83 | 678.84 | 667.68 |
|  | 6 | 709.27 | 707.11 | 704.43 | 700.69 | 690.24 |
|  | 7 | 643.24 | 708.63 | 669.44 | 625.67 | 608.56 |
|  | 8 | 571.78 | 545.73 | 585.98 | 591.65 | 579.29 |
|  | 9 | 580.04 | 611.94 | 586.8 | 559.49 | 591.29 |
|  | 10 | 555.89 | 565.17 | 546.59 | 572.87 | 571.45 |
|  | Over 11 | 424.84 | 494.34 | 430.62 | 430.26 | 472.68 |
| **Playfulness by Age when obtained (KW)** | | **Averaged total score** | **Averaged total binary score 1** | **Averaged total binary score 2** | **Averaged total binary score 3** | **Averaged total binary score 4** |
| Kruskal-Wallis H | | 34.024 | 24.583 | 31.676 | 29.847 | 19.356 |
| Mean ranks | Unweaned kitten (0 - 2 months) | 657.66 | 643.15 | 648.78 | 666.97 | 660.15 |
|  | Kitten (2 - 6 months) | 668.89 | 663.98 | 667.56 | 663.5 | 665.93 |
|  | Junior (6 months - 2 years) | 654.24 | 654.1 | 660.3 | 655.44 | 633.91 |
|  | Mature (3 – 10 years) | 500.15 | 548.6 | 511.18 | 507.86 | 534.58 |
|  | Senior (11+ years) | 307.79 | 302.67 | 303.13 | 336.38 | 428.63 |
| **Playfulness by Social Play and Fearfulness (τ)** | | **Averaged total score** | **Averaged total binary score 1** | **Averaged total binary score 2** | **Averaged total binary score 3** | **Averaged total binary score 4** |
| Social play | | 0.434 | 0.372 | 0.410 | 0.409 | 0.380 |
